# Endoplasmic Reticulum Stress Induced Synthesis of a Novel Viral Factor Mediates Efficient Replication of Genotype-1 Hepatitis E Virus

**DOI:** 10.1371/journal.ppat.1005521

**Published:** 2016-04-01

**Authors:** Vidya P. Nair, Saumya Anang, Chandru Subramani, Abhilasha Madhvi, Karishma Bakshi, Akriti Srivastava, Shalimar, Baibaswata Nayak, Ranjith Kumar CT, Milan Surjit

**Affiliations:** 1 Virology Laboratory, Vaccine and Infectious Disease Research Centre, Translational Health Science and Technology Institute, NCR Biotech Science Cluster, Faridabad, Haryana, India; 2 Department of Gastroenterology, All India Institute of Medical Sciences, Gautam Nagar, Ansari Nagar East, New Delhi, Delhi, India; Virginia Polytechnic Institute and State University, UNITED STATES

## Abstract

Hepatitis E virus (HEV) causes acute hepatitis in many parts of the world including Asia, Africa and Latin America. Though self-limiting in normal individuals, it results in ~30% mortality in infected pregnant women. It has also been reported to cause acute and chronic hepatitis in organ transplant patients. Of the seven viral genotypes, genotype-1 virus infects humans and is a major public health concern in South Asian countries. Sporadic cases of genotype-3 and 4 infection in human and animals such as pigs, deer, mongeese have been reported primarily from industrialized countries. Genotype-5, 6 and 7 viruses are known to infect animals such as wild boar and camel, respectively. Genotype-3 and 4 viruses have been successfully propagated in the laboratory in mammalian cell culture. However, genotype-1 virus replicates poorly in mammalian cell culture and no other efficient model exists to study its life cycle. Here, we report that endoplasmic reticulum (ER) stress promotes genotype-1 HEV replication by inducing cap-independent, internal initiation mediated translation of a novel viral protein (named ORF4). Importantly, ORF4 expression and stimulatory effect of ER stress inducers on viral replication is specific to genotype-1. ORF4 protein sequence is mostly conserved among genotype-1 HEV isolates and ORF4 specific antibodies were detected in genotype-1 HEV patient serum. ORF4 interacted with multiple viral and host proteins and assembled a protein complex consisting of viral helicase, RNA dependent RNA polymerase (RdRp), X, host eEF1α1 (eukaryotic elongation factor 1 isoform-1) and tubulinβ. In association with eEF1α1, ORF4 stimulated viral RdRp activity. Furthermore, human hepatoma cells that stably express ORF4 or engineered proteasome resistant ORF4 mutant genome permitted enhanced viral replication. These findings reveal a positive role of ER stress in promoting genotype-1 HEV replication and pave the way towards development of an efficient model of the virus.

## Author Summary

Hepatitis E virus (HEV) is one of the most common causes of acute and sporadic viral hepatitis. It is a small positive strand RNA virus, which is transmitted through the feco-oral route. Owing to lack of sanitation and unavailibility of safe drinking water, populations of developing and resource starved countries are prone towards HEV infection. Recent reports also indicate HEV induced acute and chronic hepatitis in organ transplant patients. Another peculiar characteristic of HEV is attributed to its ability to cause high mortality (~30%) in infected pregnant women. Even after 30 years of discovery of the virus, little information exists regarding viral life cycle and replication machinery. HEV is divided into seven genotypes. Genotype-3 and 4 viruses infect humans and a few animals (such as pigs, deer, mongeese) and have been reported from industrialized countries. Genotype-3 and 4 viruses have been successfully propagated in the laboratory in mammalian cell culture. However, genotype-1 virus, which is known to infect human and is a major public health concern in south Asian countries, replicates poorly in mammalian cell culture and no other efficient model exists to investigate the viral life cycle. With the goal of developing an efficient laboratory model of genotype-1 HEV, we attempted to identify a permissive cellular condition that would allow efficient viral replication in human hepatoma cells. Here, we report that endoplasmic reticulum stress inducing agents promote genotype-1 HEV replication by initiating cap-independent, internal translation mediated synthesis of a novel viral factor, which we have named ORF4. Further investigations revealed that ORF4 is expressed only in genotype-1 and it acts by interacting with multiple viral and host proteins and cooperates with host eEF1α1 (eukaryotic elongation factor 1 isoform 1) to control the activity of viral RNA dependent RNA polymerase. Moreover, a proteasome resistant ORF4 mutant significantly enhanced viral replication. Thus, our study identifies an optimal condition required for efficient replication of genotype-1 HEV and dissects out the molecular mechanism governing that. Data presented here will be instrumental in developing an efficient model of the virus.

## Introduction

Hepatitis E is a feco-orally transmitted positive strand RNA virus that causes acute and sporadic hepatitis in human and other animals [1]. It is also emerging to be a major cause of infection in organ transplant patients worldwide [2]. Though self-limiting in normal individuals, a peculiar characteristic of HEV is attributed to its ability to cause high mortality (~30%) in infected pregnant women [3]. The viral genome consists of a 7.2 kb 5’-capped and 3’-polyadenylated RNA, which encodes three known open reading frames (ORF); ORF1 codes for non-structural proteins, ORF2 codes for the major capsid protein and ORF3 codes for an accessory protein that associates with multiple host proteins and is supposed to modulate host signaling pathways [1]. ORF3 also interacts with host tumor susceptibility gene 101 (TSG 101) and plays an essential role in virus release [4, 5]. ORF2 has been observed to bind to the viral genomic RNA [6], induce endoplasmic reticulum (ER) stress [7, 8] and inhibit NFκB activity [9] in human hepatoma cells, suggesting a possible regulatory role of the viral capsid protein. Seven genotypes of HEV have been reported; genotype-1 (g-1), genotype-2 (g-2) exclusively infect human whereas genotype-3 (g-3), genotype-4 (g-4) infect human, pig, deer, mongeese and rabbit. Infection by genotypes 5–7 have not been reported in human. Genotype-5 (g-5), genotype-6 (g-6) infects wild boar and genotype-7 (g-7) is known to infect camel [10, 11]. Little is known about the life cycle of HEV owing to lack of a handy animal or cell culture model. Among the various genotypes, *in vitro* synthesized genome of g-3 and g-4 HEV replicates well in mammalian cell culture [12]. Attempts at achieving high replication efficiency of g-1 HEV in mammalian cell culture have not been successful [13, 14]. Interestingly, in one of the g-3 HEV infected patients, human ribosomal S17 coding sequence was found to be inserted in the ORF1 region, which conferred growth advantage to the virus [15, 16]. Molecular mechanisms underlying the above observation remain to be explored. Moreover, such an insertion appears to be a very rare occurrence.

Efficient translation and replication are two crucial events in the life of an RNA virus, tight control of which is essential for survival of both virus and its host. These events are under strict surveillance by the host defence machinery as innate antiviral measures, most common being induction of ER stress and unfolded protein response, inactivation of eukaryotic translation initiation factor 2α and shut down of cap-dependent translation [17]. Degradation of viral double stranded RNA by innate immune effectors [18] and autophagy [19] also serves as host defence mechanisms against many viruses. Viruses on the other hand, employ clever strategies to exploit the adversities imposed by the host. Innate immune response is countered by various strategies such as inhibition of type I interferon production [20], manipulation of pattern recognition receptor signaling [21], IRF3 (interferon regulatory factor 3) inhibition [22, 23] and autophagy inhibition [24]. Translational restrictions are overcome by ribosome shunting, reinitiation, stimulation of eIF4F complex assembly, inhibition of elF2α phosphorylation [25], internal ribosome entry site (IRES) mediated translation [26, 27] and execution of both cap-dependent and cap-independent modes of translation [28, 29] depending on the cellular state.

The second step in the RNA virus life cycle pertains to genome replication. Most viruses encode regulatory proteins, RNA and/or miRNA that exploit host machineries to augment viral replication. Hepatitis C, Dengue and Polio viruses activate autophagy [30, 31, 32]. Hepatitis B virus inhibits proteasome activity [33], which leads to increased viral replication. Hence, depending upon the host cellular condition, each virus seems to have evolved suitable survival strategies that permits its optimal growth.

Since g-1 HEV does not replicate efficiently in mammalian cell culture, we wondered whether any particular cellular condition might enhance viral replication. Screening of various compounds known to alter cellular condition revealed a role of ER stress inducing compounds, thapsigargin and tunicamycin in enhancing g-1 HEV replication. Further studies led to the identification of a novel viral protein synthesized from an overlapping reading frame within ORF1, which was named open reading frame 4 (ORF4). The role of ORF4 in viral replication was explored.

## Results

### Increased g-1 HEV replication by Endoplasmic Reticulum stress inducing agents

**Fig 1:**
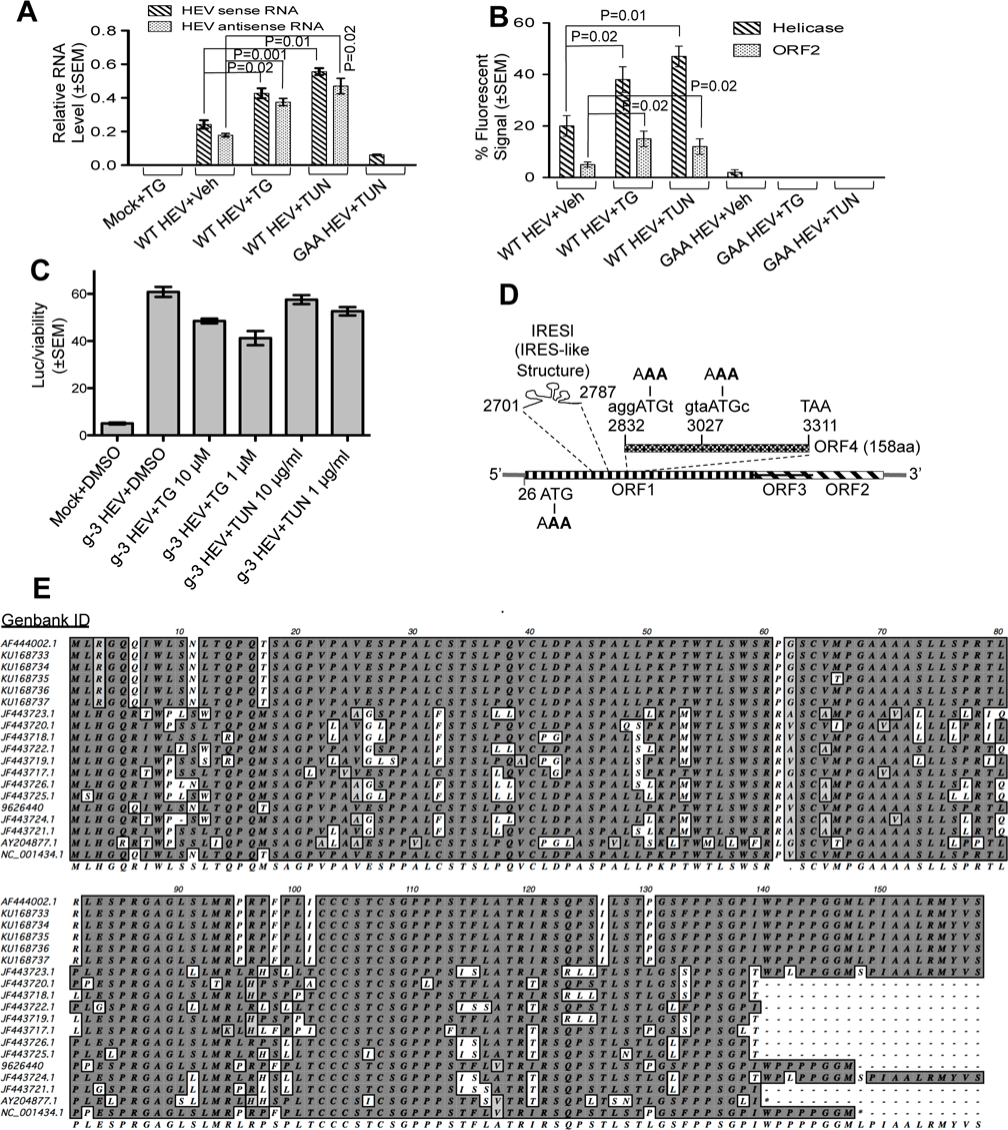
Tunicamycin and thapsigargin promote g-1 HEV replication. **(A)** QRT-PCR of wild type (WT HEV) and replication deficient (GAA HEV) HEV in Huh7 cells transfected with *in vitro* synthesized genome. TG: thapsigargin, TUN: tunicamycin. Values are mean±SEM. **(B)** Quantitation of viral ORF1 (Helicase) and ORF2 expression in Huh7 cells, transfected with wild type (WT HEV) or replication deficient (GAA HEV) HEV genomic RNA and treated with the indicated compounds. Ten random fields of approximately 30 cells in each field were counted for helicase, ORF2, DAPI (nuclear stain) fluorescence and percentage ±SEM calculated. **(C)** Measurement of secreted Gaussia luciferase activity in the culture medium of Huh7 cells expressing *in vitro* transcribed HEV genotype-3 replicon RNA and treated as indicated. Values are mean±SEM. **(D)** HEV genome organisation. Numbers indicate nucleotide position from 5’-end. Mutated bases are in bold. **(E)** ClustalW alignment of ORF4 protein sequence of indicated g-1 HEV isolates.

In order to identify the influence of a particular cellular condition on HEV replication, Huh7 cells were transfected with wild type capped genomic RNA (WT HEV). 6 days post transfection, viral replication was measured by monitoring the level of sense and antisense RNA and estimating the percentage of cells expressing viral helicase and ORF2. Note that helicase synthesis reflects ORF1 translation from genomic RNA whereas ORF2 is synthesized from the subgenomic RNA (generated after replication). A replication deficient mutant genome (GAA HEV), in which “DD” amino acids of RdRp (a.a. 1551, 1552 in ORF1) were altered to “AA”; was used to ensure specificity of both assays. Quantitative real-time PCR (QRT-PCR) of sense strand RNA level in GAA HEV transfected samples reflected the level of input RNA (quantity of transfected RNA, Fig 1A). Sense strand RNA level was approximately four fold higher in WT HEV RNA transfected sample (compared to GAA HEV), reflecting replication mediated increase. As expected, no antisense RNA was detected in GAA HEV transfected samples but was detectable at basal levels in WT HEV expressing samples. Upon treatment with known ER stress inducers; thapsigargin (TG) and tunicamycin (TUN), sense and antisense RNA levels were further increased by 2–3 fold (Fig 1A). Similarly treated samples were analyzed by immunofluorescence assay to measure the percentage of helicase and ORF2 positive cells (Fig 1B). Representative images are shown (S1 Fig). GAA HEV transfected sample contained 2% helicase positive and no ORF2 positive cells, in agreement with QRT-PCR data, confirming specificity of the assays (Fig 1B). 20% helicase and 5% ORF2 positive cells were detected in DMSO treated WT RNA transfected cells. Thapsigargin and tunicamycin treated samples contained significantly higher percentage of helicase and ORF2 positive cells in WT HEV. Helicase positive cells were absent in thapsigargin and tunicamycin treated GAA HEV sample, ruling out the possibility of increased ORF1 translation by these compounds. We next tested whether thapsigargin and tunicamycin enhanced the replication of g-3 HEV using a Gaussia luciferase secreting replicon of g-3 HEV [16]. There was no increase in luciferase level upon treatment of the replicon expressing cells with thapsigargin and tunicamycin (Fig 1C), suggesting that both compounds had a stimulatory effect only on g-1 HEV replication.

### A novel protein is expressed from an overlapping reading frame located within ORF1 of g-1 HEV *in vitro* and *in vivo*

Assuming that the mechanism underlying the observed stimulatory effect of ER stress on g-1 HEV replication is encoded in the viral genome, we analysed the g-1 HEV genome (SAR 55 strain, Genbank ID: AF444002.1) using “ATGpr”, a software to predict potential open reading frames [34]. All known ORFs of HEV were predicted. An unknown ORF of 158 amino acids within ORF1, located in +1 reading frame (with reference to ORF1, 2835–3308 bases from 5’) was also predicted, which was named ORF4 (S1 Table, Fig 1D). An ORF coding for a truncated ORF1 protein was also predicted. In contrast, sequence analysis of other HEV genotypes did not reveal any ORF resembling that of ORF4 (S1 Table). Next, we performed a bioinformatics analysis of several g-1 HEV genomic sequences available in public database to find out whether open reading frame 4 is present in all and whether the ORF4 protein sequence is conserved among the various isolates. Additionally, we analyzed the viral genomic sequence from five new cases of g-1 HEV infection, recently isolated by us at the All India Institute of Medical Sciences, New Delhi, India (Genbank ID: KU168733- KU168737, Fig 1E). All g-1 HEV genomes contain an ORF at the expected position of ORF4, with a suboptimal Kozak sequence starting either at 2832 or 2834 nucleotides, from 5’ end (Fig 1D). Three different patterns were observed with respect to termination of the putative ORF4; in 8 cases, it terminates at 3311 nucleotides (158 amino acids, full length ORF4 protein), in two cases, at 3277 nucleotides (147 amino acids) and in remaining 9 cases, it terminates at 3256 nucleotides (139 amino acids, Fig 1E). ClustalW analysis of the putative ORF4 protein sequence of these isolates revealed ~80% conservation of amino acids (Fig 1E).

**Fig 2:**
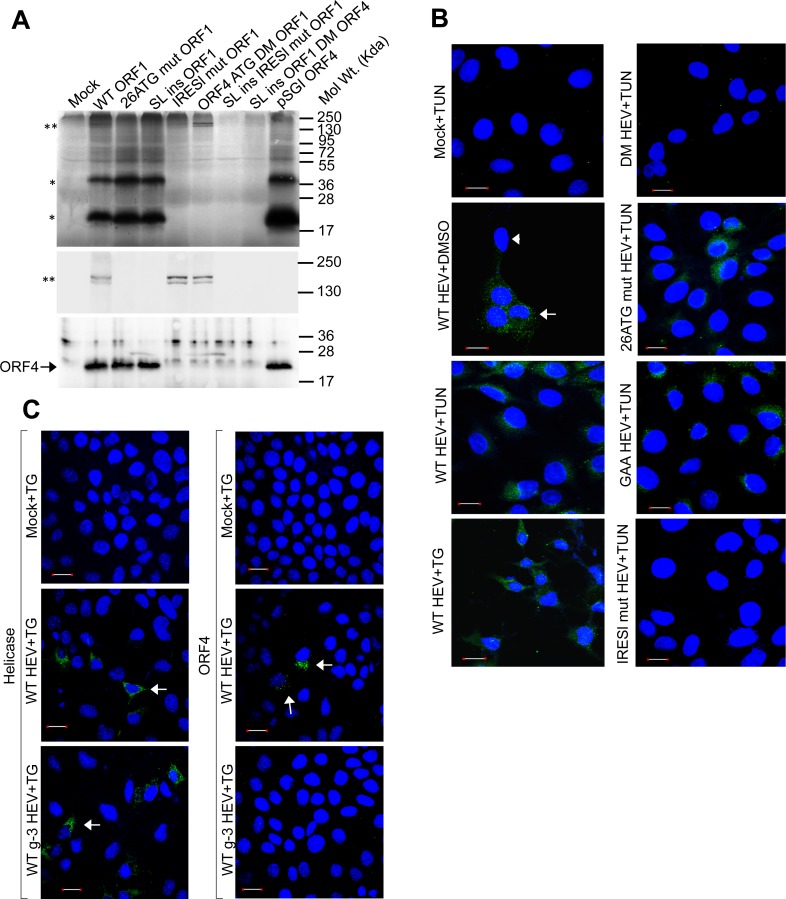
Tunicamycin and thapsigargin induce ORF4 expression. **(A)** Top: Autoradiogram showing TNT of indicated plasmids. Mock: empty vector, “**”: ORF1 protein, “*”: unknown proteins. Middle: samples from top resolved by 7% SDS-PAGE, followed by autoradiography. Bottom: samples from top resolved by 15% SDS-PAGE and western using anti-ORF4. **(B)** Immunofluorescence of ORF4 in Huh-7 cells transfected with indicated *in vitro* synthesized RNA. Scale: 20μm. Shown are merged images of nuclei (blue) and ORF4 (green). “→”: positive staining, “►”: unstained. **(C)** Immunofluorescence of Helicase and ORF4 in Huh-7 cells transfected with *in vitro* synthesized wild type HEV g-1 (WT HEV) or g-3 (WT g-3 HEV) genomic RNA. Scale: 20μm. Shown are merged images of nuclei (blue) and Helicase or ORF4 (green). “→”: positive staining.

To verify ORF4 and/or ΔORF1 expression, an *in vitro* transcription-translation assay was performed using a TNT kit. Two bands corresponding to unprocessed and probably partially processed ORF1 protein (**) were detected (Fig 2A, top and middle). Two bands corresponding to ~20kDa and ~40kDa (*) were also observed (Fig 2A, top). No such bands were detected in mock. Mutating the initiator methionine codon of ORF1 to Lysine (ATG-AAA substitution, 26 ATG mut ORF1) resulted in disappearance of ORF1 specific bands without affecting 20kDa and 40kDa bands. Similarly, blocking ORF1 translation initiation by inserting a well characterised stem loop forming sequence [35] upstream of the initiator codon of ORF1 (SL ins ORF1) abolished the bands corresponding to ORF1 without impacting 20kDa and 40kDa bands (Fig 2A). Correlating “ATGpr” prediction with above data suggested that 20kDa band may correspond to translation product of ORF4. 40kDa band could be a denaturation resistant dimeric form of ORF4 or an unrelated protein.

In agreement with the above proposition, TNT of ORF4 coding sequence produced 20 and 40 kDa bands (Fig 2A, pSGI ORF4). A peptide based rabbit polyclonal antibody was generated against the putative ORF4 protein in order to identify the unknown bands. Functionality and specificity of the antibody was validated (S2A and S2B Fig) and aliquots of TNT samples were western blotted using this antibody. Only the 20kDa band was detectable by ORF4 antibody in WT ORF1, 26 ATG mut ORF1, SL ins ORF1 and pSGI ORF4 (Fig 2A, bottom).

Two sub optimal Kozak sequences containing initiation codons are present in the ORF4 coding region (Fig 1D). Both were mutated to Lysine (ATG-AAA) in HEV ORF1 construct (ORF4 ATG DM ORF1), followed by TNT to confirm the identity of 20 and 40kDa bands. Both bands were absent in the autoradiogram and western, without affecting ORF1 level (Fig 2A). As expected, inhibiting both ORF1 and ORF4 translation initiation by stem loop insertion and ATG-AAA substitution, respectively, resulted in disappearance of all bands (SL ins ORF1 DM ORF4).

An immunofluorescence assay was conducted using ORF4 antibody to detect its expression in WT g-1 HEV genome transfected Huh7 cells. ORF4 signal was significantly higher in tunicamycin and thapsigargin treated cells compared to the DMSO control (Fig 2B). Specificity of the signal was controlled by using tunicamycin treated DM HEV (mutant g-1 HEV genome, in which ORF4 initiation codons are mutated to Lysine) transfected cells, which failed to show ORF4 signal. Tunicamycin treated 26 ATG mut HEV or GAA HEV RNA transfected cells also expressed ORF4, clearly ruling out any influence of ORF1 translation or replication on ORF4 production, respectively (Fig 2B).

In order to confirm that no ORF4 like protein is expressed in genotype-3 HEV (g-3 HEV), *in vitro* transcribed genome of a luciferase replicon of g-3 HEV (pSK HEV p6 luc) or WT g-1 HEV was transfected into Huh7 cells, followed by thapsigargin treatment and subsequent immunofluorescence staining using anti-ORF4 or anti-Helicase antibodies. Helicase expression was detectable in both samples whereas ORF4 expression was detectable only in the case of g-1 HEV (Fig 2C).

**Fig 3:**
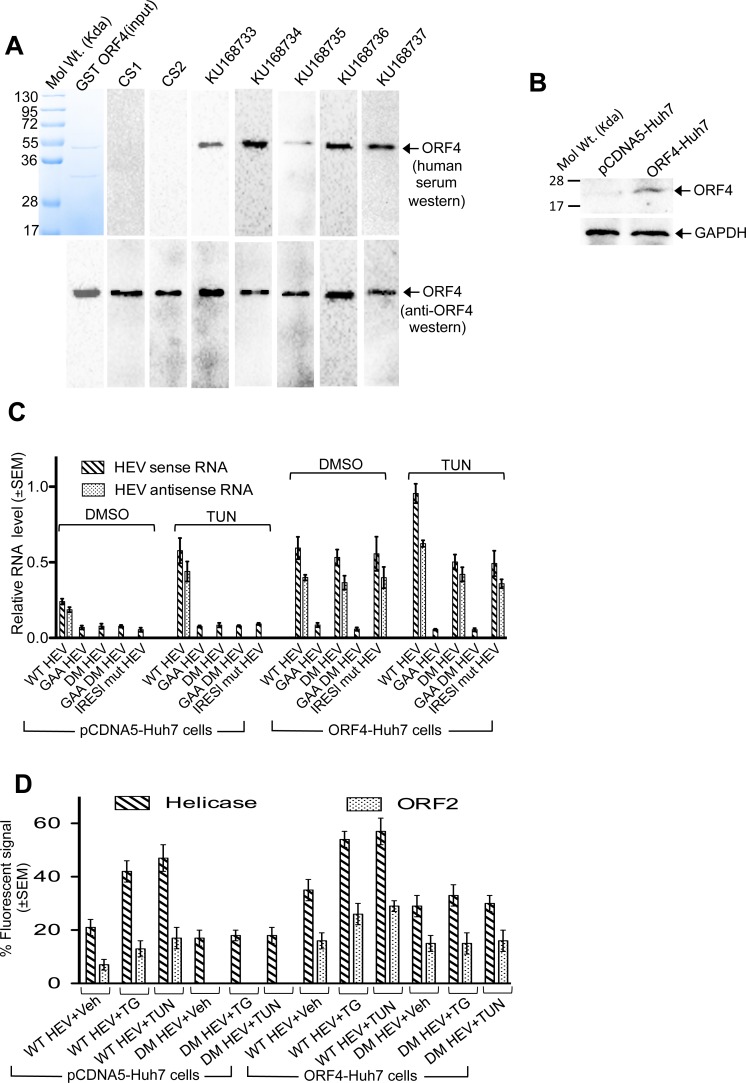
ORF4 antibody is detected in HEV patients and its over expression enhances viral replication. **(A)** Top: Purified GST-ORF4 stained with coomassie blue (left most) and western of equal aliquots of the same using healthy (CS1, CS2) and acute HEV infected (KU168733-KU168737) patient sera. Bottom: Top blots reprobed with anti-ORF4 antibody. **(B)** Western of whole cell extract from indicated cells using ORF4 and GAPDH antibodies. **(C)** QRT-PCR of sense and anti-sense RNA in pCDNA5-Huh7 and ORF4-Huh7 cells transfected with *in vitro* synthesized wild type (WT) or mutant HEV genome and treated as indicated.**(D)** Quantitation of viral ORF1 (helicase) and ORF2 expression in pCDNA5-Huh7 and ORF4-Huh7 cells, transfected with wildtype (WT HEV) or ORF4 expression deficient mutant (DM HEV) HEV genomic RNA and treated with the indicated compounds. Ten random fields of approximately 30 cells in each field were counted for helicase, ORF2, DAPI (nuclear stain) fluorescence and percentage ±SEM calculated.

Next, we analysed ORF4 expression in the five g-1 HEV infected patients, in which ORF4 sequence was conserved (KU168733-KU168737, Fig 1E). ORF4 expression was assessed indirectly by monitoring the level of anti-ORF4 antibody, if any. Purified GST-ORF4 protein was readily detected by serum from all 5 patients (KU168733-KU168737) whereas serum from two healthy (CS1-CS2) individuals were negative (Fig 3A).

### Expression of ORF4 is essential for basal and tunicamycin dependent increase in HEV replication

A stable cell line of Huh7 constitutively expressing Flag-tagged ORF4 was generated (ORF4-Huh7) to explore the role of ORF4 in HEV replication (Fig 3B). WT HEV or GAA HEV genome was transfected into ORF4-Huh7 and its control (pCDNA5-Huh7). The level of sense and antisense RNA of WT HEV was approximately two fold higher in DMSO treated ORF4-Huh7 cells compared to control (Fig 3C). As expected, GAA HEV mutant was unable to replicate. Tunicamycin treatment increased sense and antisense RNA by two fold in control and four fold in ORF4-Huh7 cells. DM HEV behaved like GAA HEV in control cells in the presence and absence of tunicamycin. In contrast to GAA HEV, DM HEV produced both sense and antisense RNA at levels equivalent to WT HEV in DMSO treated ORF4-Huh7 cells and remarkably, these levels remained unaltered in the presence of tunicamycin (Fig 3C). Similar pattern was obtained in immunofluorescence analysis of helicase and ORF2 positive cells (Fig 3D). Thapsigargin too displayed a pattern similar to tunicamycin (Fig 3D).

### Internal initiation of ORF4 translation, driven by an Internal ribosome entry site-like (IRESl) element located within ORF1

**Fig 4:**
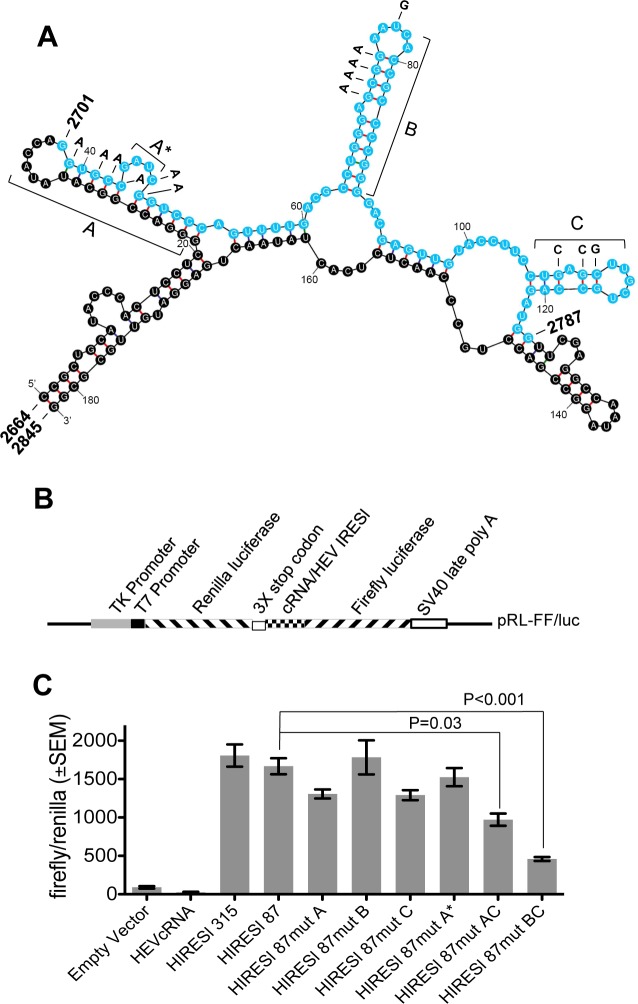
An IRESl (Internal ribosome entry site-like) element located upstream of ORF4 coding sequence drives its translation independent of ORF1. **(A)** Predicted secondary structure of IRESl using “mfold”. Cyan letters indicate core IRESl sequence. A, B, C: stem loops, A*: bulge. Substitutions that impair IRESl are in bold. **(B)** Organization of Dual luciferase reporter plasmid. **(C)** Dual luciferase reporter assay. Values are mean±SEM.

ORF1 translation is cap-dependent [1]. However, ORF4 could be translated in the absence of cap-dependent translation (SL ins ORF1, Fig 2A). Considering its location deep inside ORF1, we wondered whether ORF4 synthesis was driven by an internal translation initiation mechanism. Bioinformatics analysis of viral RNA flanking ORF4 region using “Reg RNA” [36] indicated the presence of a putative IRES-like element between 2701–2787 bases (Fig 1D, IRESl). Analysis of same sequence using “IRESite” [37] predicted weak homology with Equine Rhinitis A virus-1 IRES [38]. Secondary structure analysis of 2664–2845 bases encompassing the predicted IRES-like element using “mfold” [39] revealed the presence of three stem loops within 2701–2787 bases (Fig 4A, sequence in cyan). Increase in sequence length (315 bases, 2619–2933 bases) did not alter those stem loops, indicating their stability (S3 Fig). A dual luciferase reporter assay was conducted to evaluate the functionality of the IRES-like element by placing it between Renilla and Firefly coding sequences (Fig 4B). Three consecutive stop codons were introduced downstream of the Renilla coding sequence to ensure termination of cap-dependent translation of Renilla luciferase. 315 bases from HEV genome encompassing the IRES-like element (HIRESl 315) or 468 bases from 3501–3968 nucleotides (negative control for background Firefly activity, HEVcRNA) were inserted downstream of Renilla, preceding the Firefly start site (Fig 4B). Measurement of the Firefly and Renilla luciferase ratios revealed a significantly higher Firefly activity in HIRESl 315 sample (Fig 4C). The core IRES-like element (2701–2787 bases, HIRESl 87) also displayed similar activity (Fig 4C). Next, several mutant constructs were generated in which individual stem loops were destroyed by altering a few nucleotides at a time. Impairing stem loops A, B, C or bulge (A*) did not affect Firefly activity. A moderate and high reduction in Firefly activity was seen in samples containing dual mutations of both A,C and B,C stem loops, respectively (Fig 4C).

Dual mutations of both B and C were introduced into plasmids containing HEV ORF1 and HEV genome (IRESl mut ORF1 and IRESl mut HEV, respectively). In TNT of IRESl mut ORF1 construct, ORF4-specific band disappeared without affecting that of ORF1 (Fig 2A). No ORF4 was detected in cells transfected with IRESl mut HEV RNA upon tunicamycin treatment (Fig 2B). Expectedly, IRESl mut HEV genome replication was significantly reduced irrespective of tunicamycin treatment in pCDNA5-Huh7 cells, which could be restored in ORF4-Huh7 cells, though in a tunicamycin insensitive manner (Fig 3C).

### ORF4 directly and indirectly associates with several viral proteins and promotes the assembly of a multi protein complex

**Fig 5:**
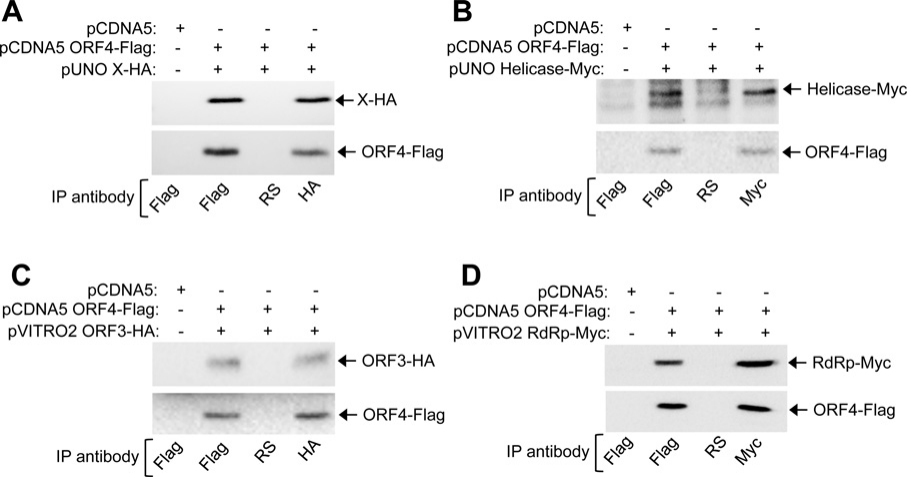
ORF4 interacts with multiple viral proteins. **(A)** CoIP of ORF4 and X expressing Huh7 extract, immunoprecipitated and revealed using indicated antibodies. RS: Rabbit preimmune serum. **(B)** CoIP of ORF4 and Helicase expressing Huh7 extract, immunoprecipitated and revealed using indicated antibodies. **(C)** CoIP of ORF4 and ORF3 expressing Huh7 extract, immunoprecipitated and revealed using indicated antibodies. **(D)** CoIP of ORF4 and RdRp expressing Huh7 extract, immunoprecipitated and revealed using indicated antibodies.

**Fig 6:**
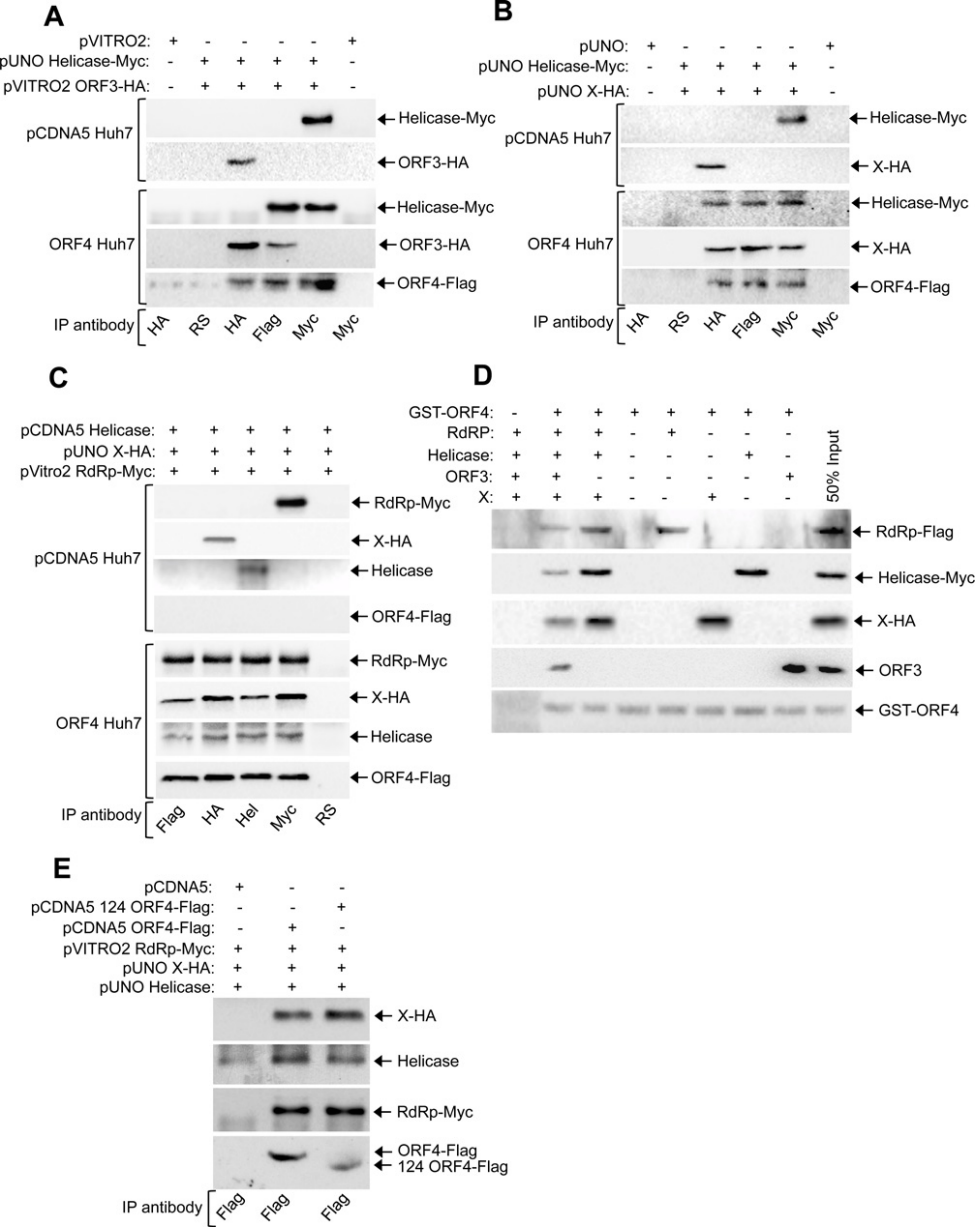
ORF4 mediates the assembly of a protein complex consisting of viral RdRp, Helicase and X. **(A)** CoIP and western of pCDNA5-Huh7 and ORF4-Huh7 stable cell line extract transiently expressing myc-tagged helicase and HA-tagged ORF3 proteins, immunoprecipitated and revealed using indicated antibodies. RS: rabbit pre immune serum. **(B)** CoIP and western of pCDNA5-Huh7 and ORF4-Huh7 stable cell line extract transiently expressing myc-tagged helicase and HA-tagged X proteins, immunoprecipitated and revealed using indicated antibodies. **(C)** CoIP and western of pCDNA5-Huh7 and ORF4-Huh7 stable cell line extract transiently expressing helicase, X and RdRp, immunoprecipitated and revealed using indicated antibodies. **(D)** Pull down of ORF4 interacting proteins using GST-ORF4 as bait, revealed using indicated antibodies. Lane 1 contains GST as bait. **(E)** Pull down of full length ORF4 (pCDNA5 ORF4-Flag) and 1-124aa mutant ORF4 (pCDNA5 124 ORF4-Flag) interacting proteins using ORF4-Flag as bait, revealed using indicated antibodies.

To explore the mechanism(s) by which ORF4 stimulated viral replication, we identified its interaction partners among viral proteins. ORF4 directly interacted with helicase, X and ORF3 proteins of g-1 HEV, evident from Yeast Two Hybrid (Y2H) assay (Table 1). X protein of g-3 HEV also interacted with ORF4, however neither g-3 helicase nor g-3 ORF3 interacted with ORF4 (Table 1). Using overlapping deletions of ORF4, the interaction domain was mapped to 54–122 amino acids for X and ORF3 and 1–124 amino acids for helicase protein of g-1 HEV (Table 2). Coimmunoprecipitation (CoIP) of Huh7 cells transfected with plasmids encoding ORF4 and various g-1 HEV proteins confirmed its interaction with X, helicase and ORF3 (Fig 5A, 5B and 5C). Interestingly, CoIP also demonstrated that ORF4 interacted with g-1 RdRp in Huh7 cells (Fig 5D). No other viral proteins interacted with ORF4 in CoIP (S4 Fig). Since X and ORF3 interacted with the same region of ORF4 and helicase appeared to interact with a broader region/multiple domains of ORF4, we next determined whether X and ORF3 competed or cooperated with helicase for binding to ORF4. In ORF4-Huh7 cells coexpressing helicase and ORF3, though ORF4 associated with helicase and ORF3 and vice versa, ORF3 was not coprecipitated with helicase, indicating that all three were not in the same complex (Fig 6A). However, helicase, X and ORF4 could be coprecipitated, indicating cooperativity among them (Fig 6B).

**Table 1:** ORF4 directly associates with multiple viral proteins: Yeast Two Hybrid analysis of ORF4 interaction partners in g-1 HEV.

YEAST COTRANSFORMANTS	LT^-^	LTHA^-^	LTH^-^A^+^	LT^-^ + X-α-gal	LTH^-^+3AT(mM)		
					5	10	20
Y2H GOLD	-	-	-	-	-	-	-
BD-ORF4+AD	+++	-	-	-	-	-	-
BD-ORF4+AD-ORF3	+++	+++	+++	+++	+	-	-
BD-ORF4+AD-X	+++	+++	+++	+++	+++	++	+
BD-ORF4+AD-Helicase	+++	+++	++	++	+	-	-
BD-ORF4+AD-Methyl transferase	+++	-	-	-	-	-	-
BD-ORF4+AD-Protease	+++	-	-	-	-	-	-
BD-ORF4+AD-Y domain	+++	-	-	-	-	-	-
BD-ORF4+AD-V domain	+++	-	-	-	-	-	-
BD-ORF4+AD-D domain	+++	-	-	-	-	-	-
BD-ORF4+AD-RdRp	+++	-	-	-	-	-	-
BD-ORF4+AD-ORF2	+++	-	-	-	-	-	-
BD+AD-ORF3	+++	-	-	-	-	-	-
BD+AD-X	+++	-	-	-	-	-	-
BD+AD-Helicase	+++	-	-	-	-	-	-
BD-ORF3+AD-TSG101	+++	+++	+++	+++	+++	++	+
BD-ORF4+AD-g-3 ORF3	+++	+	+	-	-	-	-
BD-ORF4+AD-g-3 X	+++	+++	+++	+++	+++	++	+
BD-ORF4+AD-g-3 Helicase	+++	-	-	-	-	-	-

LT^-^: Leucine, Tryptophan deficient medium; LTHA^-^: Leucine, Tryptophan, Histidine and Adenine Hemisulphate deficient medium; LTH^-^A^+^: Leucine, Tryptophan, Histidine deficient medium supplemented with Aureobasidin; LT^-^ + X-α-gal: Leucine, Tryptophan deficient medium supplemented with X-α-gal; 3AT: 3-amino 1,2,4- Triazole. +++ Strong growth, ++ average growth, + poor growth,—no growth. BD: Binding domain, AD: Activation domain. Data represents the summary of 3 independent experiments.

**Table 2:** Deletion mapping of interaction domains in ORF4 for binding with g-1 ORF3, X and Helicase.

YEAST COTRANSFORMANTS	LT^-^	LTHA^-^	LTH^-^A^+^	LT^-^ + X-α-gal	LTH^-^+3AT(mM)		
					5	10	20
AD-(1–158) ORF4 + BD-ORF3	+++	+++	+++	+++	+	-	-
AD-(1–41) ORF4 + BD-ORF3	+++	-	-	-	-	-	-
AD-(1–61) ORF4 + BD-ORF3	+++	-	-	-	-	-	-
AD-(1–124) ORF4 + BD-ORF3	+++	+++	+++	+++	-	-	-
AD-(54–105) ORF4 + BD-ORF3	+++	-	-	-	-	-	-
AD-(54–122) ORF4 + BD-ORF3	+++	+++	+++	++	-	-	-
AD-(76–122) ORF4 + BD-ORF3	+++	-	-	-	-	-	-
AD-(1–158) ORF4 + BD-X	+++	+++	+++	+++	+++	++	+
AD-(1–41) ORF4 + BD-X	+++	-	-	-	-	-	-
AD-(1–61) ORF4 + BD-X	+++	-	-	-	-	-	-
AD-(1–124) ORF4 + BD-X	+++	+++	+++	+++	+++	++	+
AD-(54–105) ORF4 + BD-X	+++	-	-	-	-	-	-
AD-(54–122) ORF4 + BD-X	+++	+++	+++	+++	+	-	-
AD-(76–122) ORF4 + BD-X	+++	-	-	-	-	-	-
AD-(1–158) ORF4 + BD-Helicase	+++	+++	++	++	+	-	-
AD-(1–41) ORF4 + BD-Helicase	+++	-	-	-	-	-	-
AD-(1–61) ORF4 + BD-Helicase	+++	-	-	-	-	-	-
AD-(1–124) ORF4 + BD-Helicase	+++	+++	+	+	-	-	-
AD-(54–105) ORF4 + BD-Helicase	+++	-	-	-	-	-	-
AD-(54–122) ORF4 + BD-Helicase	+++	++	-	-	-	-	-
AD-(76–122) ORF4 + BD-Helicase	+++	-	-	-	-	-	-

Numbers in parentheses indicate coordinates of amino acids of ORF4 (from N-terminus) cloned in the AD vector. Abbreviations are as in Table 1. Data represents the summary of 3 independent experiments.

We next tested whether viral RdRp associated with the X-helicase-ORF4 complex. CoIP in ORF4-Huh7 and its control cells demonstrated that RdRp coprecipitated with X and helicase only in the presence of ORF4 (Fig 6C). Moreover, all four appeared to be part of one complex as helicase and RdRp antibody could coprecipitate X and ORF4 and vice versa (Fig 6C). A pull down assay using purified proteins further confirmed that ORF4 indeed mediated the assembly of a complex consisting of RdRp, helicase, X and ORF4 (Fig 6D). In contrast, ORF3 inhibited assembly of the above complex, probably by competing for binding to ORF4 (Fig 6D, compare lane 2 with 3).

Since many of the g-1 HEV isolates encode a truncated ORF4 protein consisting of 139aa or 147aa (from N-terminus) and our Y2H based mapping of the X, Helicase and ORF3 interaction region of ORF4 was found to be confined to N-terminal 124aa, a pull down assay was performed using a deletion mutant of full length ORF4 protein comprising of N-terminal 124aa (124 ORF4-Flag). As expected, 124 ORF4-Flag could assemble a complex consisting of RdRp, X and Helicase; similar to the full length ORF4 protein (Fig 6E). These data suggest that ORF4 is functionally active in all g-1 isolates.

### ORF4 enhances the activity of viral RNA dependent RNA polymerase (RdRp)

**Fig 7:**
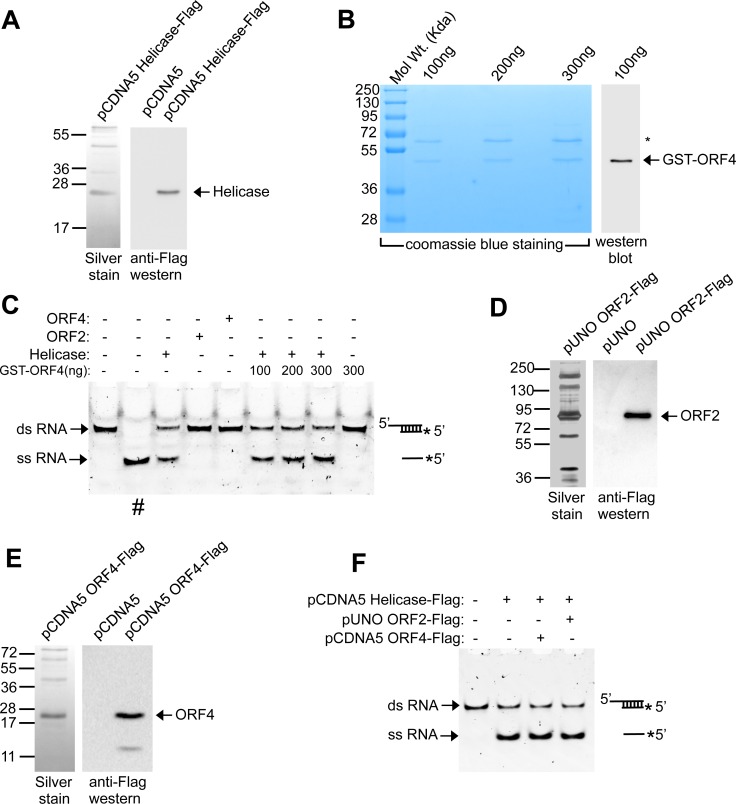
ORF4 does not affect RNA strand displacement ability of g-1 HEV Helicase. **(A)** Left panel: SDS-PAGE and Silver staining of Flag antibody immunoprecipitated samples from Huh7 cells transiently transfected with pCDNA5 Helicase-flag vector. Right panel: Western of pCDNA5 and pCDNA5 Helicase-flag vectors using anti-Flag antibody. **(B)** Left panel: SDS PAGE and Coomassie blue staining of indicated amount of purified bacterial GST-ORF4 protein. “*” indicates copurified unrelated protein band. Right panel: Western of 100ng purified GST-ORF4 protein using anti-ORF4 antibody. **(C)** Assay of RNA strand displacement by Helicase in the presence of GST-ORF4. “#”: heat denatured dsRNA. **(D)** Left panel: SDS-PAGE and Silver staining of Flag antibody immunoprecipitated samples from Huh7 cells transiently transfected with pUNO ORF2-flag vector. Right panel: Western of pUNO and pUNO ORF2-flag vector using anti-Flag antibody. **(E)** Left panel: SDS-PAGE and Silver staining of Flag antibody immunoprecipitated samples from Huh7 cells transiently transfected with pCDNA5 ORF4-flag vector. Right panel: Western of pCDNA5 and pCDNA5 ORF4-flag vector using anti-Flag antibody. **(F)** RNA strand displacement assay demonstrating the effect of Huh7 cell expressed ORF4 on HEV helicase enzymatic activity. Schematic at the right represents the structure of dsRNA and ssRNA.

Since ORF4 interacts with helicase and RdRp, we wondered whether it influenced their activities. A helicase assay using Huh7 purified Helicase-Flag (Fig 7A) and bacterial purified GST-ORF4 (Fig 7B) revealed that ORF4 had no effect on RNA unwinding activity of helicase (Fig 7C). Huh7 purified ORF2-Flag (Fig 7D) was used as a negative control. Helicase assay in the presence of Huh7 purified ORF4 (Fig 7E) produced similar results (Fig 7F), indicating that under our experimental conditions, ORF4 had no effect on dsRNA unwinding activity of viral helicase.

**Fig 8:**
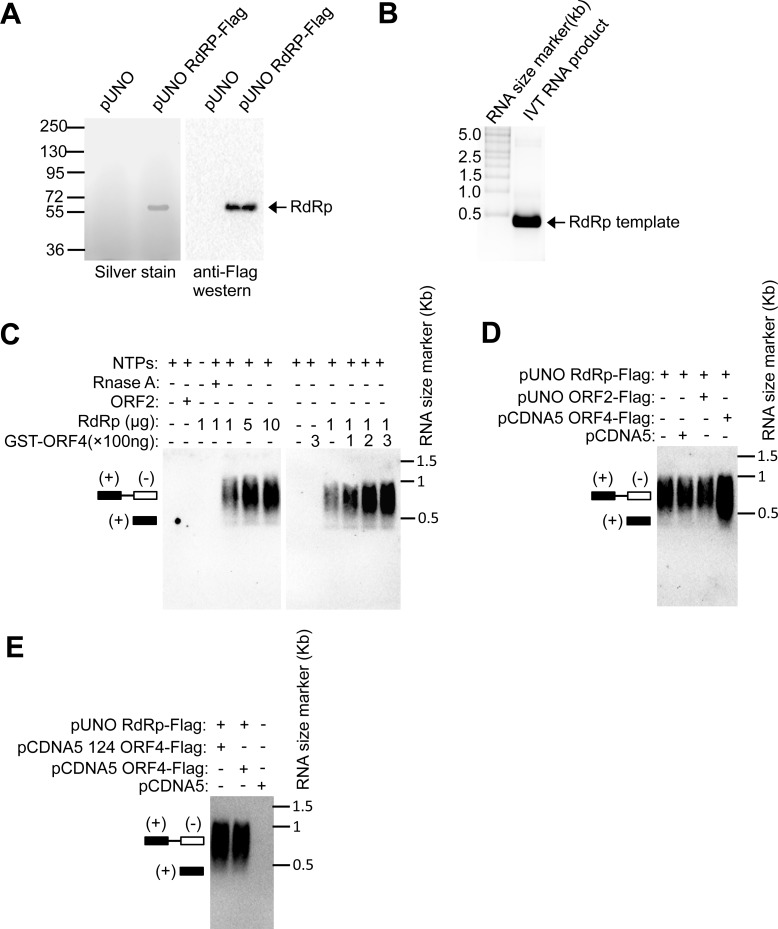
ORF4 promotes viral RdRp activity. **(A)** Left panel: SDS-PAGE and Silver staining of Flag antibody immunoprecipitated samples from Huh7 cells transiently transfected with pUNO empty vector or pUNO RdRp-flag vector. Right panel: Western of left panel samples using anti-Flag antibody. **(B)** Formaldehyde Agarose gel electrophoresis followed by staining with Ethidium bromide to detect *in vitro* transcribed RNA (IVT RNA product) used as template in RdRp assay. **(C)** RdRp assay using purified RdRp and GST-ORF4. Schematic illustrates position of dsRNA (+,-) and ss RNA. NTPs: Nucleotide triphosphate mix. **(D)** RdRp assay using purified RdRp, ORF2 and ORF4 proteins. **(E)** RdRp assay using purified RdRp, ORF2 and 124 ORF4 (1-124aa) proteins.

Next, an RdRp assay was performed using Huh7 purified RdRp-Flag (Fig 8A) in the presence of increasing amount of bacterial purified GST-ORF4 or ORF4-Flag. An *in vitro* transcribed RNA containing 130 bases from 5’-end and 210 bases from 3’-end of g-1 HEV genome was used as template for the assay (Fig 8B). Addition of ORF4 significantly increased double stranded RNA intermediate level (680 bases), reflecting enhanced RdRp activity (Fig 8C). Observed activity was specific to viral RdRp because no signal was obtained in reactions containing ORF2-Flag or GST-ORF4 alone. RdRp assay in the presence of Huh7 cell purified full length ORF4-Flag or 124 ORF4-Flag (1–124 amino acids of ORF4) produced a similar effect (Fig 8D and 8E) confirming that both full length and 124 aa ORF4 indeed enhanced viral RdRp activity.

### Eukaryotic Translation elongation factor 1α isoform1 (eEF1α1) mediates the association between ORF4 and RdRp, absence of which impairs the stimulatory effect of ORF4 on g-1 RdRp activity

ORF4 indirectly associated with g-1 RdRp. In an independent study carried out in our laboratory to isolate direct interacting partners of g-1 HEV RdRp by screening a human fetal brain cDNA library using Yeast Two Hybrid (Y2H) technique, 21 host proteins were identified (Table 3). We tested the ability of those proteins to associate with ORF4. Only eukaryotic translation elongation factor 1 α isoform 1 (eEF1α1), Tubulin beta (Tubβ) and actin gamma isoform 1 were found to be common interaction partners of both RdRp and ORF4 (Table 3). Though eEF1α1 interacted with equal strength with both RdRp and ORF4, Tubβ and Actin gamma 1 weakly interacted with ORF4, compared to RdRp (Table 3, compare growth on 3-amino 1,2,4 triazole).

**Table 3:** Yeast Two Hybrid screening of host factors that directly interact with g-1 RdRp and ORF4.

YEAST COTRANSFORMANTS	LT^-^	LTHA^-^	LTH^-^A^+^	LT^-^+X-α-gal	LTH^-^+3AT(mM)		
					5	10	**20**
Y2H GOLD	-	-	-	-	-	-	-
BD-RdRp+AD	+++	++	+	+	+	-	-
BD-RdRp+AD-COG1	+++	+++	+++	+++	+++	++	+
BD-RdRp+AD-RTN1	+++	+++	++	+++	+++	++	+
BD-RdRp+AD-EEF1A1	+++	+++	+++	+++	++	+	+
BD-RdRp+AD-TUBB	+++	+++	+++	+++	+++	+++	+++
BD-RdRp+AD-ZDHHC6	+++	+++	+++	+++	+++	++	+
BD-RdRp+AD-MACF1	+++	+++	+++	+++	+++	+++	++
BD-RdRp+AD-TMEM66	+++	+++	+++	+++	+++	++	++
BD-RdRp+AD-ACTG1	+++	+++	+++	+++	+++	+++	+++
BD-RdRp+AD-SLC2A1	+++	+++	+++	+++	+++	+++	++
BD-RdRp+AD-MAP1B	+++	+++	+++	+++	+++	+++	++
BD-RdRp+AD-PSAP	+++	+++	+++	+++	+++	+++	++
BD-RdRp+AD-BTB6	+++	+++	+++	+++	+++	+++	+++
BD-RdRp+AD-FEZ1	+++	+++	+++	+++	+++	++	+
BD-RdRp+AD-H2AFY2	+++	+++	+++	+++	+++	++	+
BD-RdRp+AD-PDCL	+++	+++	+++	+++	++	++	+
BD-RdRp+AD-LSAMP	+++	+++	+++	+++	+++	+++	++
BD-RdRp+AD-APLP1	+++	+++	+++	+++	++	++	+
BD-RdRp+AD-MARCKSL1	+++	+++	+++	+++	++	++	+
BD-RdRp+AD-CIRBP	+++	+++	+++	+++	++	++	+
BD-RdRp+AD-RNF187	+++	+++	+++	+++	++	++	+
BD-RdRp+AD-ARM2	+++	+++	+++	+++	++	++	+
BD-ORF4+AD	+++	-	-	-	-	-	-
BD-ORF4+AD-COG1	+++	-	-	-	-	-	-
BD-ORF4+AD-RTN1	+++	-	-	-	-	-	-
BD-ORF4+AD-EEF1A1	+++	+++	+++	+++	++	++	-
BD-ORF4+AD-TUBB	+++	+++	+++	+++	+	-	-
BD-ORF4+AD-ZDHHC6	+++	+++	+++	-	-	-	-
BD-ORF4+AD-MACF1	+++	+++	+++	-	-	-	-
BD-ORF4+AD-TMEM66	+++	-	-	-	-	-	-
BD-ORF4+AD-ACTG1	+++	+++	+++	+++	+	-	-
BD-ORF4+AD-SLC2A1	+++	++	+	-	-	-	-
BD-ORF4+AD-MAP1B^a^	+++	+++	++	++	+++	++	+
BD-ORF4+AD-PSAP^b^	+++	+++	+	+	-	-	-
BD-ORF4+AD-BTB6^b^	+++	+++	+	+	-	-	-
BD-ORF4+AD-FEZ1	+++	+	+	-	-	-	-
BD-ORF4+AD-H2AFY2	+++	-	-	-	-	-	-
BD-ORF4+AD-PDCL	+++	-	-	-	-	-	-
BD-ORF4+AD-LSAMP	+++	-	-	-	-	-	-
BD-ORF4+AD-APLP1	+++	-	-	-	-	-	-
BD-ORF4+AD-MARCKSL1	+++	-	-	-	-	-	-
BD-ORF4+AD-CIRBP	+++	-	+	-	-	-	-
BD-ORF4+AD-RNF187	+++	-	-	-	-	-	-
BD-ORF4+AD-ARM2^b^	+++	+++	+	+	-	-	-
BD+AD-COG1	+++	-	-	-	-	-	-
BD+AD-RTN1	+++	-	-	-	-	-	-
BD+AD-EEF1A1	+++	-	-	-	-	-	-
BD+AD-TUBB	+++	-	-	-	-	-	-
BD+AD-ZDHHC6	+++	-	-	-	-	-	-
BD+AD-MACF1	+++	-	-	-	-	-	-
BD+AD-TMEM66	+++	-	-	-	-	-	-
BD+AD-ACTG1	+++	-	-	-	-	-	-
BD+AD-SLC2A1	+++	-	-	-	-	-	-
BD+AD-MAP1B^a^	+++	++	++	++	+++	++	+
BD+AD-PSAP	+++	-	-	-	-	-	-
BD+AD- BTB6	+++	-	-	-	-	-	-
BD+AD-FEZ1	+++	-	-	-	-	-	-
BD+AD-H2AFY2	+++	-	-	-	-	-	-
BD+AD-PDCL	+++	-	-	-	-	-	-
BD+AD-LSAMP	+++	-	-	+	-	-	-
BD+AD-APLP1	+++	-	-	+	-	-	-
BD+AD-MARCKSL1	+++	-	-	+	-	-	-
BD+AD-CIRBP	+++	+	-	+	-	-	-
BD+AD-RNF187	+++	-	-	-	-	-	-
BD+AD-ARM2	+++	-	-	+	-	-	-

Y2H gold strain was transformed in indicated combinations and plated on media lacking Leucine, Tryptophan (LT^-^). Eight random colonies from each transformants were replica plated to media containing various selection markers, as indicated and their growth monitored over a period of four days. Abbreviations are as in Table 1. ^a^false positive. ^b^very weak interaction, considered insignificant. Data represents the summary of 3 independent experiments.

**Fig 9:**
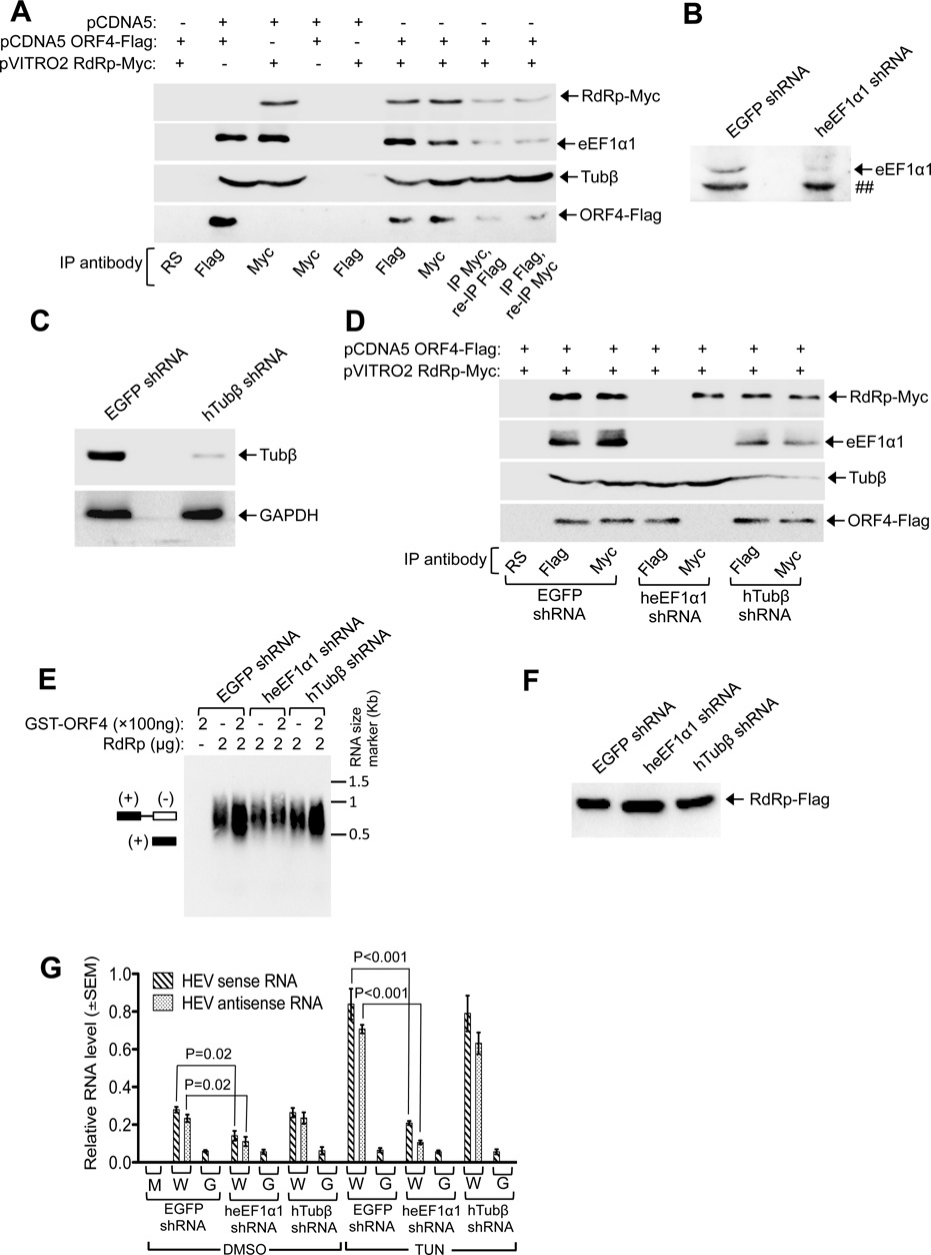
eEF1α1 bridges the interaction between ORF4 and RdRp. **(A)** CoIP of RdRp, ORF4, eEF1α1 and Tubulin β (TUBβ) in Huh7 cells. **(B)** Western of Huh7 cells expressing EGFP or heEF1α1 shRNA. “##”: nonspecific band. **(C)** Western of Huh7 cells expressing EGFP or hTubβ shRNA (Top). Bottom: same blot reprobed with anti-GAPDH. **(D)** CoIP of Huh7 cells expressing EGFP, heEF1α1 and hTubβ shRNA. **(E)** HEV RdRp assay using EGFP, heEF1α1 and hTubβ shRNA expressing cells. **(F)** Anti-Flag western of Flag-affinity purified g-1 RdRp protein from Huh7 cells expressing g-1 RdRp-Flag and shRNAs against EGFP, eEF1α1 and Tubβ. **(G)** QRT-PCR of sense and antisense RNA of WT HEV (W) and GAA HEV (G) in Huh7 cells expressing EGFP, heEF1α1, hTubβ shRNA. M: Mock.

CoIP of Huh7 cells expressing RdRp and ORF4 demonstrated that both eEF1α1 and Tubβ associated with ORF4 and RdRp (Fig 9A). We could not detect actin gamma 1 association with ORF4 in CoIP (S4 Fig, top panel). Fraction of both eEF1α1 and Tubβ appeared to associate with ORF4-RdRp complex because both of them could be detected in samples subjected to two rounds of sequential immunoprecipitation (Fig 9A). Next, eEF1α1 and Tubβ proteins were ablated using shRNA to find out whether either or both bridged the interaction between ORF4 and RdRp. shRNAs were approximately 95% and 80% effective in reducing eEF1α1 and Tubβ protein, respectively (Fig 9B and 9C). CoIP revealed the inability of ORF4 to associate with RdRp in the absence of eEF1α1 though association of ORF4 and RdRp with Tubβ remained unaffected (Fig 9D). In contrast, ablation of Tubβ had no effect on the interaction between ORF4, RdRp and eEF1α1 (Fig 9D).

Next, an RdRp assay was conducted using purified RdRp-Flag from respective shRNA expressing cells. Stimulatory effect of ORF4 on RdRp activity was absent in samples lacking eEF1α1 (Fig 9E). Level of RdRp in Flag-affinity purified sample was verified by western and quantified to ensure that eEF1α1 or Tubβ ablation did not prevent RdRp translation (Fig 9F). Finally, we measured the level of sense and antisense RNA of wild type (W) and GAA mutant (G) HEV in DMSO or tunicamycin treated Huh7 cells expressing EGFP, heEF1α1 or hTubβ shRNA. Lack of eEF1α1 significantly reduced the level of both RNAs in DMSO and tunicamycin treated samples, confirming its essential role in g-1 HEV replication (Fig 9G) whereas absence of Tubβ had no effect.

### Proteasome resistant ORF4 mutant promotes replication of g-1 HEV genome

**Fig 10:**
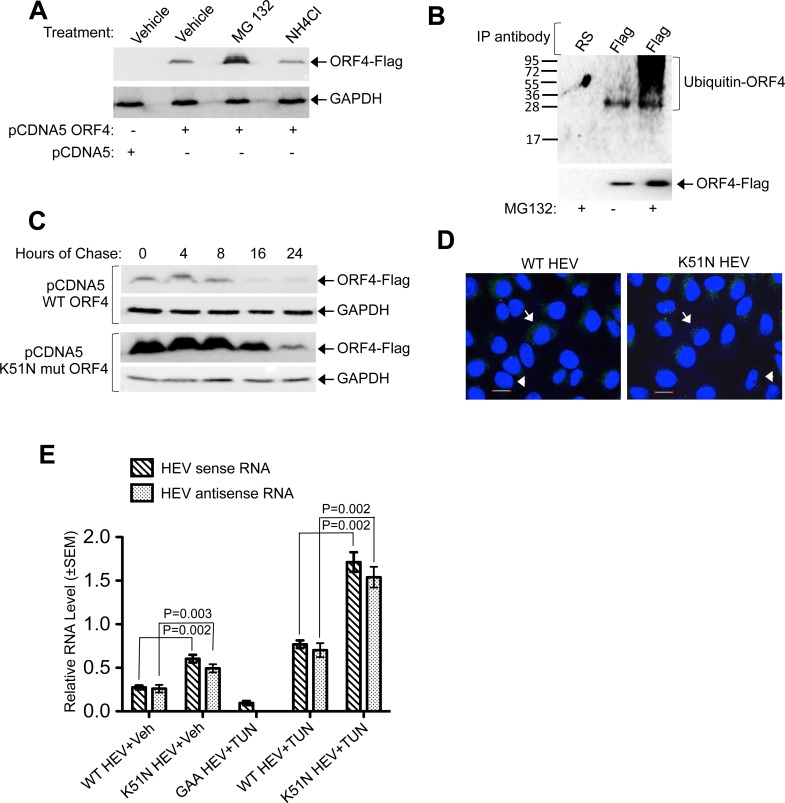
ORF4 is a target of host ubiquitin-proteasome machinery. **(A)** Western blot using anti-Flag (top) and anti-GAPDH (bottom). **(B)** Western blot using anti-ubiquitin (top) and anti-Flag (bottom). **(C)** Anti-Flag western blot of Huh7 cells expressing WT and K51N mut ORF4, treated with cycloheximide (panel 1, 3). Same blots were reprobed with anti-GAPDH (panel 2, 4). **(D)** Immunofluorescence of ORF4 in Huh7 cells transfected with *in vitro* synthesized WT HEV or K51N HEV. Scale: 20μm. Shown are merged images of nuclei (blue) and ORF4 (green). “→”: positive staining, “►”: unstained. **(E)** QRT-PCR of HEV sense and anti-sense RNA from Huh7 cells transfected and treated as indicated. Veh: Vehicle, TUN:tunicamycin.

Measurement of the level of ORF4 protein in the presence of proteasomal inhibitor MG132 and lysosomal acid protease inhibitor NH_4_Cl revealed its sensitivity to the former (Fig 10A). Degradation of ORF4 by proteasome was further evident from its polyubiquitination status (Fig 10B). ORF4 contains a lysine at 51^st^ amino acid position flanked by two proline residues (hydrophobic amino acids), which represents a putative ubiquitination site. This Lysine was mutated to Asparagine (K51N mut ORF4). Monitoring the level of wild type and K51N mut ORF4 in the presence of cycloheximide (blocks de novo translation) revealed significantly higher stability of the mutant (Fig 10C), confirming that ORF4 is indeed a target of the proteasome.

The K51N substitution was introduced into g-1 HEV genome, followed by transfection of mutant genome into Huh7 cells. Immunofluorescence analysis revealed an increase in the number of ORF4 positive cells in the K51N mutant (K51N HEV, Fig 10D). Measurement of sense and antisense RNA in wild type and mutant genome transfected cells revealed higher level of both RNAs in the K51N mutant, indicative of enhanced replication of proteasome resistant ORF4 encoding genome, which was further increased upon tunicamycin treatment (Fig 10E).

Since a proteasome resistant ORF4 mutant genome could significantly enhance the viral replication, we wondered whether such mutations are prevalent in natural cases of g-1 HEV infection. Analysis of the ORF4 ubiquitination site in g-1 HEV sequences illustrated in Fig 1E revealed that 51^st^ Lysine is absolutely conserved in all. However, in one case (AY204877.1), 50^th^ and 52^nd^ Proline residues were substituted with Serine and Leucine, respectively (Fig 1E). In 6 cases (JF443721.1- JF443726.1), 50^th^ Proline was substituted with Leucine (Fig 1E). The above substitutions are supposed to prevent ubiquitination at 51^st^ Lysine. Bioinformatics analysis of the above seven ORF4 protein sequences using “UbPred” software (predicts potential ubiquitination sites in a protein [40]) also indicated lack of ubiquitination at the 51^st^ Lysine. Thus, viruses containing these sequences should produce a proteasome resistant ORF4 protein, similar to K51N mutation.

### g-3 and g-1 RdRp significantly differ in their ability to associate with host and viral proteins

**Fig 11:**
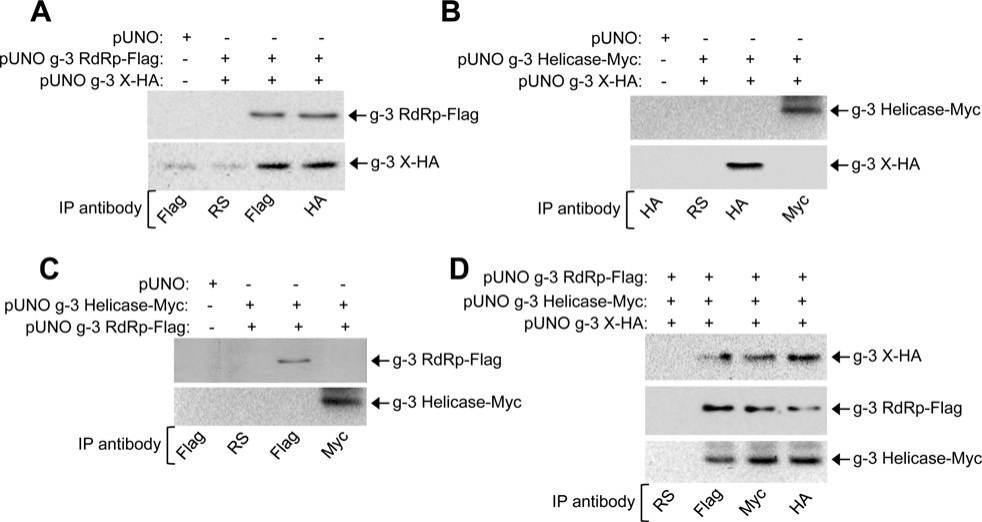
G-3 RdRp, X and Helicase associate with each other in Huh7 cells. **(A)** CoIP and western of Huh7 cell extract transiently expressing g-3 RdRp and X, immunoprecipitated and revealed using indicated antibodies. **(B)** CoIP and western of Huh7 cell extract transiently expressing g-3 Helicase and X, immunoprecipitated and revealed using indicated antibodies. **(C)** CoIP and western of Huh7 cell extract transiently expressing g-3 RdRp and Helicase, immunoprecipitated and revealed using indicated antibodies. **(D)** CoIP and western of Huh7 cell extract transiently expressing g-3 RdRp, Helicase and X, immunoprecipitated and revealed using indicated antibodies.

Despite lacking ORF4, g-3 HEV replicates better than g-1 virus in mammalian cell culture [12]. We hypothesised that some host protein(s) might be substituting the function of ORF4 in the g-3 virus, allowing it to bypass the dependency on ER stress dependent synthesis of ORF4. Host proteins identified as g-1 RdRp interaction partners (Table 3) were tested for their ability to associate with g-3 RdRp (Table 4). Only 14 out of 21 g-1 RdRp interaction partners associated with g-3 RdRp (Table 4), indicating that g-3 RdRp interaction profile is different from that of its g-1 counterpart. Therefore, it may interact with additional host proteins that did not interact with g-1 RdRp. We further tested the direct and indirect interactions of g-3 RdRp with other proteins of g-3 HEV by Y2H and CoIP assays. No intra-viral interaction partner of g-3 RdRp could be detected in Y2H assay, in agreement with the data obtained for g-1 RdRp (S2 Table). However, CoIP of Huh7 cell extract expressing both g-3 RdRp and g-3 X revealed that both of them coprecipitated with each other, indicating an interaction between them (Fig 11A). No interaction was observed between g-3 RdRp and helicase or g-3 X and helicase (Fig 11B and 11C). Next, a CoIP assay was performed to assess whether g-3 RdRp, X and helicase could assemble a complex. Indeed, all three could be coprecipitated (Fig 11D), indicating that they remain associated with each other. These findings also suggest that some host factor is essential for bridging the interaction between g-3 RdRp, X and helicase.

**Table 4:** Yeast Two Hybrid screening of host factors that directly interact with g-3 HEV RdRp.

YEAST COTRANSFORMANTS	LT^-^	LTHA^-^	LTH^-^A^+^	LT^-^+X-α-gal	LTH^-^+3AT(mM)			PPI status
					5	10	20	
Y2H GOLD	-	-	-	-	-	-	-	
BD-g-3 RdRp+AD	+++	+	++	-	+	-	-	
BD-g-3 RdRp+AD-COG1	+++	++	++	-	+	-	-	N
BD-g-3 RdRp+AD-RTN1	+++	+	+++	+	+	-	-	N
BD-g-3 RdRp+AD-EEF1A1	+++	+++	+++	+	+++	+++	++	Y
BD-g-3 RdRp+AD-TUBB	+++	+++	+++	+	+++	+++	+++	Y
BD-g-3 RdRp+AD-ZDHHC6	+++	+++	+++	+	+++	+++	++	Y
BD-g-3 RdRp+AD-MACF1	+++	+++	+++	+	+++	+++	++	Y
BD-g-3 RdRp+AD-TMEM66	+++	++	+++	+	++	+	-	Y
BD-g-3 RdRp+AD-ACTG1	+++	+++	+++	+	+++	+++	+++	Y
BD-g-3 RdRp+AD-SLC2A1	+++	+++	+++	+	+++	++	++	Y
BD-g-3 RdRp+AD-MAP1B	+++	+++	+++	++	+++	+++	++	Y
BD-g-3 RdRp+AD-PSAP	+++	+++	+++	+	+++	+++	+++	Y
BD-g-3 RdRp+AD-BTB6	+++	+++	+++	++	+++	+++	+++	Y
BD-g-3 RdRp+AD-FEZ1	+++	+++	+++	+	+++	+++	++	Y
BD-g-3 RdRp+AD-H2AFY2	+++	+++	+++	+	+++	+++	++	Y
BD-g-3 RdRp+AD-PDCL	+++	+	++	+	+	-	-	N
BD-g-3 RdRp+AD-LSAMP	+++	+	++	+	+	-	-	N
BD-RdRp+AD-APLP1	+++	+	++	+	+	-	-	N
BD-g-3 RdRp+AD-MARCKSL1	+++	+	++	+	++	+	-	N
BD-g-3 RdRp+AD-CIRBP	+++	++	++	+	+	-	-	N
BD-g-3 RdRp+AD-RNF187	+++	++	++	+	++	+	-	Y
BD-g-3 RdRp+AD-ARM2	+++	+++	++	+	+++	++	+	Y
BD+AD-COG1	+++	-	-	-	-	-	-	
BD+AD-RTN1	+++	-	-	-	-	-	-	
BD+AD-EEF1A1	+++	-	-	-	-	-	-	
BD+AD-TUBB	+++	-	-	-	-	-	-	
BD+AD-ZDHHC6	+++	-	-	-	-	-	-	
BD+AD-MACF1	+++	-	-	-	-	-	-	
BD+AD-TMEM66	+++	-	-	-	-	-	-	
BD+AD-ACTG1	+++	-	-	-	-	-	-	
BD+AD-SLC2A1	+++	-	-	-	-	-	-	
BD+AD-MAP1B	+++	++	++	++	+++	++	+	
BD+AD-PSAP	+++	-	-	-	-	-	-	
BD+AD- BTB6	+++	-	-	-	-	-	-	
BD+AD-FEZ1	+++	-	-	-	-	-	-	
BD+AD-H2AFY2	+++	-	-	-	-	-	-	
BD+AD-PDCL	+++	-	-	-	-	-	-	
BD+AD-LSAMP	+++	-	-	+	-	-	-	
BD+AD-APLP1	+++	-	-	+	-	-	-	
BD+AD-MARCKSL1	+++	-	-	+	-	-	-	
BD+AD-CIRBP	+++	+	-	+	-	-	-	
BD+AD-RNF187	+++	-	-	-	-	-	-	
BD+AD-ARM2	+++	-	-	+	-	-	-	

Y2H gold strain was transformed in indicated combinations and plated on media lacking Leucine, Tryptophan (LT^-^). Eight random colonies from each transformants were replica plated to media containing various selection markers, as indicated and their growth monitored over a period of four days. Abbreviations are as in Table 1. Y: Positive, N: Negative. PPI status: Protein-protein interaction status. Data represents the summary of 3 independent experiments.

## Discussion

The current study attempts to address a long standing issue for researchers in HEV biology, pertaining to poor replication of g-1 HEV in cell culture. We show through multiple experiments that a previously unknown viral protein, which we have named ORF4, is essential for proper functioning of RdRp of g-1 HEV. Because ORF4 is synthesized only under condition of ER stress and it is a short-lived protein, replication of viral genome is inefficient in normal cells. Thus, it appears that ER stress, which is probably initiated as an antiviral response by the host, turns out to be the ideal cellular condition for optimal replication of g-1 HEV. Such a mechanism seems to be remarkably suited to the life of the virus, given that individuals under stress such as pregnant women display enhanced sensitivity towards HEV infection. These findings also suggest that a diverse range of diseases which induce hepatic ER stress, may sensitize individuals towards g-1 HEV infection. A study involving clinical assessment of hepatic stress, quantitation of ORF4 expression and viral titre in liver biopsy of different categories of g-1 HEV patients may unravel the correlation between ER stress, degree of ORF4 expression and disease severity.

Interestingly, ORF4 is encoded only by the g-1 HEV. Our bioinformatics analysis did not predict the presence of ORF4 in other HEV genotypes and experimental analysis of g-3 HEV replicon ruled out the possibility of ORF4 expression by g-3 HEV. Moreover, ER stress inducing compounds tunicamycin and thapsigargin did not have any effect on g-3 HEV replication. The above observations gave rise to two important questions: (a) Does ORF4 really play an important role during the natural course of g-1 HEV replication, (b) If ORF4 is indispensible for g-1 HEV replication, how do other genotypes of HEV replicate in its absence. To answer the first question, we analysed all available g-1 HEV genome sequences and not only observed the presence of ORF4 but also observed a very high level of conservation of ORF4 protein sequence among all g-1 HEV isolates (see Fig 1E). Though the C-terminal 19 amino acids of ORF4 were absent in ~50% of the viral genomes, our experimental data demonstrate that these 19 amino acids are dispensible for known functions of ORF4. The N-terminal 124 amino acids of ORF4 are sufficient for interacting with other viral and host proteins (see Fig 6E) and *in vitro*, it is able to stimulate RdRp activity just like the full length ORF4 (see Fig 8E). Therefore, ORF4 seems to be indispensable for g-1 HEV life cycle.

Since our study indicates that ORF4 most likely acts by interacting with multiple viral and host proteins to assemble a replication complex and promotes g-1 RdRp activity by interacting with host eEF1α1, in order to understand how other genotypes of HEV replicate in the absence of ORF4, we compared the protein interaction profile of g-1 RdRp with that of g-3 RdRp. Though g-1 and g-3 RdRp share ~85% homology at the amino acid level, only 14 out of 21 g-1 RdRp interacting host proteins could interact with g-3 RdRp. Moreover, g-3 RdRp interacted with g-3 X protein in Huh7 cells, indicating that some host protein(s) bridges that interaction. Further, g-3 RdRp, X and helicase assembled a complex, probably mediated by some host protein(s). G-3 X and g-3 helicase may display differential interaction profile than their g-1 counterparts, as observed in the case of g-3 RdRp. Thus, it is worth speculating that host proteins substitute the function of ORF4 in the case of g-3 HEV. Host protein interaction profile of g-3 RdRp, X and helicase needs to be established to identify the host proteins involved in assembling g-3 RdRp, X and helicase complex. Nevertheless, our data provides evidence for the acquisition of a regulatory system that enhances replication in g-1 HEV as it is not seen in any other genotype. ER stress independent constitutive assembly of the viral replication complex might account for the observed better replication efficiency of g-3 virus in mammalian cell culture.

Investigation of the mechanism(s) driving ORF4 synthesis revealed that it is independent of ORF1 translation, which is cap-dependent. Subsequent studies led to the discovery of a RNA regulatory element, which mediated cap-independent translation of ORF4. *In vitro* as well as in dual luciferase reporter assays, the HEV regulatory element functioned efficiently irrespective of thapsigargin and tunicamycin treatment. However, it was active only under conditions of ER stress in its natural location in the HEV genome, probably because the regulatory element is inaccessible or remains bound to inhibitory factors in the absence of ER stress. Several other viral and cellular IRESs are known to be active only under specific conditions. Notably, an IRES within Human immunodeficiency Virus-1 mediates viral structural protein synthesis during G2/M phase of cell cycle [27] and under conditions of oxidative stress [41]. Human cytomegalovirus latency protein pUL138 is translated by an IRES like element during serum stress [28]. A subset of cellular mRNAs such as c-Myc, Bip, Apaf-1, p53 and XIAP are translated through IRESs under conditions of stress, hypoxia and/or in a cell cycle dependent manner [42].

Though our data demonstrates the coexistence of both cap-dependent and cap-independent modes of translation in g-1 HEV, we have designated the RNA regulatory element as “IRES-like” element because it does not closely resemble other well known IRESs except for weak homology with the ERAV-1 IRES. Identification of IRES trans acting factors and detailed understanding of the mechanism of translation mediated by this element would confirm whether it is a bonafide IRES. Nonetheless, current data adds g-1 HEV to the list of RNA viruses where both cap-dependent and independent modes of translation coexist.

eEF1α1 ablation inhibited basal RdRp activity and antisense RNA synthesis whereas Tubβ ablation had no effect. Stimulatory effect of ORF4 on RdRp activity was also dependent on the level of eEF1α1, indicating crucial role of the latter in viral replication. Notably, eEF1α1 is important for replication and encapsidation of many plant and animal RNA viruses [43]. It binds to RdRp of Tobacco mosaic virus and silencing it inhibits infection [44]. eEF1α1 interacts with p33 protein of Tombus virus and this interaction is essential for viral antisense RNA synthesis [45]. eEF1α1 appears to have a similar role in g-1 HEV antisense RNA synthesis.

Our data demonstrates that ORF4 is degraded by the host proteasome. Early in life, virus focuses on replication and later on switches towards release of progeny. As lack of ORF4 dampens RdRp activity, it is possible that ORF4 performs two important functions in the life of g-1 HEV. At early phase, it promotes viral replication and later on, being a short lived protein, it acts as a regulatory switch to shift from replication to release. On the contrary, proteasomal degradation of ORF4 might also be an anti-viral strategy evolved in the host to restrict virus spread. Our limited analysis involving sequence analysis of 19 g-1 HEV isolates demonstrated conservation of the 51^st^ Lysine. However, the ubiquitination site was lost in 7 sequences owing to alteration of 50^th^ Proline to Leucine, suggesting that viruses in those patients produced a proteasome resistant ORF4. It is noteworthy that 5 out of the 7 sequences were isolated from fulminant hepatic failure (FHF) patients and 2 were acute viral hepatitis patients. However, considering the very limited number of samples, it might be a coincidence that majority of them represented FHF cases. Experimental analysis of replication efficiency of these 7 genomes will further substantiate the role of proteasome resistant ORF4 in g-1 HEV replication. Furthermore, an elaborate study involving more patient samples should be conducted to confirm the above observation. Analysis of correlation between disease severity and appearance of stabilizing (proteasome resistant) mutations in the ORF4 would further establish its role as a pro-viral factor. Identification of stabilizing mutations in ORF4 seems to have an important practical application in developing a more efficient model of HEV. HEV genome harbouring the ORF4 K51N mutation, which displays a high replication efficiency, might be expressed in cell lines stably expressing viral capsid and ORF3 protein to generate a robust system for producing HEV in the laboratory.

In conclusion, the present study provides yet another example of an opportunistic pathogen, which transforms the adversities imposed by the host towards its own benefit. Identification of ORF4 as an essential proviral factor, which is expressed only under conditions of ER stress, likely explains the inability of g-1 HEV to replicate efficiently in mammalian cell culture under standard laboratory condition. A proteasome resistant ORF4 harbouring HEV genome will be useful for establishing an efficient model of g-1 HEV. Our study also suggests that different HEV genotypes may have evolved different molecular mechanisms to exploit the host and successfully complete their life cycles.

## Materials and Methods

### Plasmids, viral RNA, cell culture, transfection and shRNA expression

HEV ORFs were PCR amplified from pSKHEV2 (genbank: AF444002.1) or pSK HEV p6 luc (genbank: JQ679013.1) plasmids and cloned into the required vectors following standard protocols [46]. HEV genomic RNA was *in vitro* synthesised, as described [13]; size and integrity was monitored by formaldehyde agarose gel electrophoresis. Huh7 human hepatoma cells were as described in Surjit et al. [4] and it was originally obtained from the laboratory of C.M. Rice [47]. HEK 293T cells were obtained from ATCC (USA). Cells were maintained in Dulbecco’s modified Eagle medium (DMEM) containing 10% Fetal Calf Serum (FCS), 50 I.U./mL Penicillin and Streptomycin, in 5% CO_2_. Cells were transfected using Lipofectamine 2000 or 3000, following manufacturer’s protocol (Life Technologies, USA) or electroporated. shRNAs were designed using Oligoengine 2.0 software for cloning into pSUPER puro vector, following manufacturer’s guidelines (Oligoengine, USA). Additional details in supplementary methods.

### Antibodies and reagents

Antibodies against Flag, GAPDH, Myc, Ubiquitin and actin gamma were from Santa Cruz Biotechnology (USA). Antibodies against HA, eEF1α1, Tubulin β were from Sigma (USA). MG132, thapsigargin, tunicamycin, cycloheximide and NH_4_Cl were from Sigma (USA). Rabbit polyclonal antibodies against HEV ORF2, Helicase and ORF4 were synthesised at Genscript (USA) and validated in our lab (See supplementary methods). All chemicals were added 24 hours post transfection and maintained for 16 hours, or as indicated. Effective concentrations: MG132-25μM; cycloheximide-100μg/ml; thapsigargin-1μM; tunicamycin-10μg/ml; NH_4_Cl-30μM.

### Analysis of HEV patient sample

HEV infected, acute liver failure serum samples were obtained from patients registered in the liver clinic of Department of Gastroenterology, All India Institute of Medical Sciences, New Delhi, India. Serum was also collected from two healthy individuals with informed consent. A total 57 samples were tested for anti HEV IgM by ELISA and viral RNA with nested semi quantitative RT-PCR and quantitative real time PCR (primer sequences in S3 Table), respectively. Five samples were HEV IgM and g-1 HEV RNA positive. ORF4 coding and flanking region of these samples were sequenced and data submitted to genbank (ID: KU168733-KU168737). For testing cross-reactivity with purified ORF4 protein in western, serum was diluted 1:5000, followed by incubation with 1:5000 diluted goat anti-human IgG HRPO (Southern Biotech, USA). Viral genomic sequence of patient samples were aligned to HEV sequence (AF444002.1) for comparison.

### ClustalW alignment

ORF4 coding region nucleotide sequence of different HEV isolates was obtained from Genbank and translated into protein sequence using MacVector software. ClustalW alignment was done using MacVector. AF444002.1 sequence was considered as reference.

### Isolation of total RNA and quantitative real time PCR (QRT-PCR) assay

Total RNA was isolated using TRI reagent (MRC, USA), followed by reverse transcription (RT) and QRT-PCR, as described [48]. Random hexamers and HEVAS RP oligo were used in RT for detecting sense and antisense strands, respectively. Primer sequences are provided in S3 Table.

### Immunofluorescence assay (IFA), immunoprecipitation (IP) and western blotting (WB)

Done as described [4]. Goat anti rabbit alexa Fluor 488 (Molecular probes, USA) secondary antibody was used in IFA. Nucleus was stained with 4’ 6’- diamino-2-phenylindole (Antifade gold, Molecular probes). Images were acquired using a 60X objective in a confocal microscope (Olympus FV1000) and analyzed by Fluoview software. Details in supplementary methods.

### *In vitro* coupled transcription-translation (TNT)

A T7 polymerase based TNT kit (Promega, USA) was used for *in vitro* synthesis of proteins, following manufacturer’s instructions.

### Yeast two hybrid assay

A GAL4 based system (Clontech, USA) was used following manufacturer’s instructions. Briefly, Y2H gold strain was transformed using lithium acetate with required BD and AD plasmids, followed by replica plating of 8 random transformants on different selection media to evaluate the activity of reporters. ORF3-TSG 101 interaction was used as a positive control [4].

For screening the Y2H cDNA library of human fetal brain, g-1 RdRp was cloned into pGBKT7 vector and its self activation potential was evaluated in Y2H gold strain (S4A Table). A mate and plate human fetal brain cDNA library (Clontech, USA) was used to screen the interaction partners of g-1 RdRp, following the instructions of the manufacturer. Mating condition and efficiency is mentioned (S4B Table). From evaluation of the diploids obtained after mating to identification of the bonafide interaction partners is summarized (S4C Table). All interactions were confirmed by retransformation of the prey and bait plasmids in pair along with appropriate negative controls (Table 3).

### Luciferase reporter and cell viability assay

Dual luciferase reporter constructs (Firefly and Renilla, 1μg/well) were transfected into HEK 293T cells at 70% confluency in 48 well plate using lipofectamine 2000. Compounds were added for 16 hours, followed by luciferase assay using Dual Luciferase reporter assay kit (Promega, USA). Firefly luciferase values were divided by that of renilla and plotted. Gaussia luciferase was measured from culture medium using renilla luciferase assay kit (Promega, USA). Viability of same cells were measured using stable tetrazolium salt WST-1 (Roche, USA). Gaussia values were normalised to that of cell viability and plotted. Values are mean ± SEM of three independent experiments done in triplicate.

### Protein purification

GST-ORF4 was expressed in *E*. *Coli C-41(DE3)* strain (0.1 mM IPTG, 18°C, 16 hours). Soluble protein was bound to Glutathione Sepharose beads, washed and eluted using 20mM glutathione. Eluted protein was Flag-affinity purified following manufacturer’s instructions (Sigma, USA). Final protein was recovered in PBS. Flag-tagged ORF2, helicase, RdRp and ORF4 were purified from Huh7 cells transiently expressing respective proteins by Flag-affinity purification. Silver staining was done using Pierce silver stain kit (Thermo Scientific, USA).

### Pull down assay

Glutathione Sepharose bound GST-ORF4 was mixed with equal amount of purified RdRp, X, Helicase and ORF3 in CoIP buffer [20mM Tris (pH 7.4), 150mM NaCl, 1mM EDTA (pH 8.0), 1mM EGTA (pH 8.0), 1% Triton X 100, 2.5mM Sodium Pyrophosphate, 1mM β glycerol phosphate, 1mM sodium orthovanadate, protease inhibitor cocktail] and rotated overnight at 4°C. Beads were washed thrice in same buffer, bound proteins eluted in 20mM glutathione, followed by western blotting using indicated antibodies.

### RNA strand displacement assay

Done as described [49] with the modification that the 16 base RNA oligo was labelled with 6 FAM (6-carboxyfluorecein). Additional details in supplementary methods.

### RNA dependent RNA polymerase (RdRp) assay

Done as described [50] with the modification that DIG-II-UTP was used. Additional details in supplementary methods.

### Statistics

Data are presented as mean ± SEM of at least three independent experiments, analyzed using ‘‘GraphPad Prism” by the Student t test. p < 0.05 was considered significant.

### Ethics statement

Peripheral Blood samples were obtained from HEV infected and healthy adults with informed consent. Written consent was obtained from each individual. The study protocol was approved by the Ethics committee of All India Institute of Medical Sciences, New Delhi, India.

## Supporting Information

S1 Fig Thapsigargin and tunicamycin induce HEV helicase and ORF2 expression. Immunofluorescent visualization of Helicase and ORF2 expression in Huh7 cells expressing wild type (WT) and replication defective mutant (GAA HEV) HEV genome and treated with vehicle (DMSO), thapsigargin (Tg) or tunicamycin (Tun). Goat anti-rabbit alexa fluor-488 secondary antibody was used for all. Nuclei stained with DAPI.
(TIF)

S2 Fig Validation of ORF4, helicase and ORF2 antibodies. **(A)** Immunofluorescent visualization of ORF4 (top), Helicase (middle) and ORF2 (bottom) protein in Huh7 cells expressing respective plasmids and stained with indicated primary antibodies. “**→**” represents positive staining and “►”represents unstained cells. Goat anti-rabbit alexa fluor-488 secondary antibody was used for all. Nuclei stained with DAPI. RS: Rabbit pre immune serum. **(B)** Western of Huh7 whole cell extract transfected with pCDNA5 or pCDNA5 ORF4 plasmids using anti-Flag and anti-ORF4 antibodies, as indicated. **(C)** Western of Huh7 whole cell extract transfected with pCDNA5 or pCDNA5 Helicase plasmids using anti-Flag and anti-Helicase antibodies, as indicated. **(D)** Western of Huh7 whole cell extract transfected with pUNO or pUNO ORF2 plasmids using anti-Flag and anti-ORF2 antibodies, as indicated.
(TIF)

S3 Fig HEV genome encodes an IRES-like element. Secondary structure prediction of 2619–2933 bases (from 5’-end) of HEV genome using “mfold”.
(TIF)

S4 Fig Identification of interaction partners of g-1 RdRp by CoIP assay. Mock (pCDNA5) or pCDNA5 ORF4 along with indicated g-1 viral protein (VP, such as Methyltransferase, ORF2, PCP, Y domain and V domain) transfected Huh7 cells were immunoprecipitated with anti-ORF4 antibody, followed by western blotting using the indicated antibodies. 25% of the pCDNA5 ORF4+VP transfected samples used for immunoprecipitation were loaded as whole cell extract (WCE). In the case of actin gamma CoIP (top panel), only pCDNA5 ORF4 was transfected.
(TIF)

S1 Table Analysis of HEV genome using “ATGpr”. Summary of “ATGpr” analysis of HEV genomes.
(DOCX)

S2 Table Identification of intra-viral interaction partners of g-3 RdRp by Yeast Two Hybrid assay. Summary of Yeast Two Hybrid assay using g-3 RdRp as bait and other g-3 viral proteins as prey.
(DOCX)

S3 Table Primers and oligos used in various experiments. List of oligo sequences used in various experiments.
(XLSX)

S4 Table Screening of a human fetal brain cDNA Yeast Two Hybrid library to identify the host interaction partners of g-1 RdRp. Summary of the Y2H cDNA library screening process.
(DOCX)

S1 File Supplementary methods. Detailed procedure of all cloning steps and other methodologies are provided in S1 File.
(DOCX)
