# Supplementary figures and images for: Endoplasmic Reticulum Stress Induced Synthesis of a Novel Viral Factor Mediates Efficient Replication of Genotype-1 Hepatitis E Virus
Source: PLoS Pathog. 2016 Apr 1;12(4):e1005521. doi: 10.1371/journal.ppat.1005521 (PMC4817972; doi:10.1371/journal.ppat.1005521)

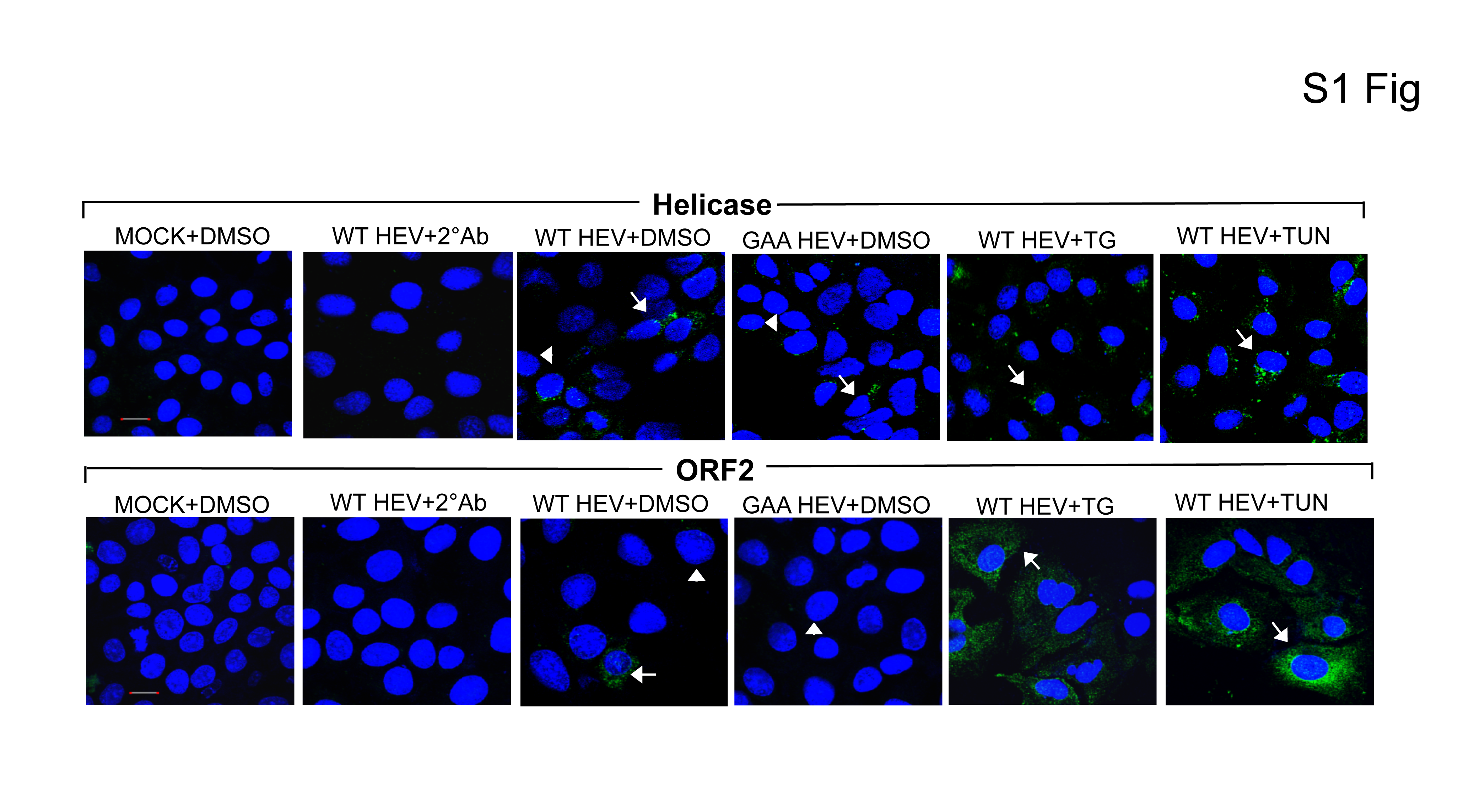

Supplement: S1 Fig — Immunofluorescent visualization of Helicase and ORF2 expression in Huh7 cells expressing wild type (WT) and replication defective mutant (GAA HEV) HEV genome and treated with vehicle (DMSO), thapsigargin (Tg) or tunicamycin (Tun). Goat anti-rabbit alexa fluor-488 secondary antibody was used for all. Nuclei stained with DAPI. (TIF) [file ppat.1005521.s001.tif]

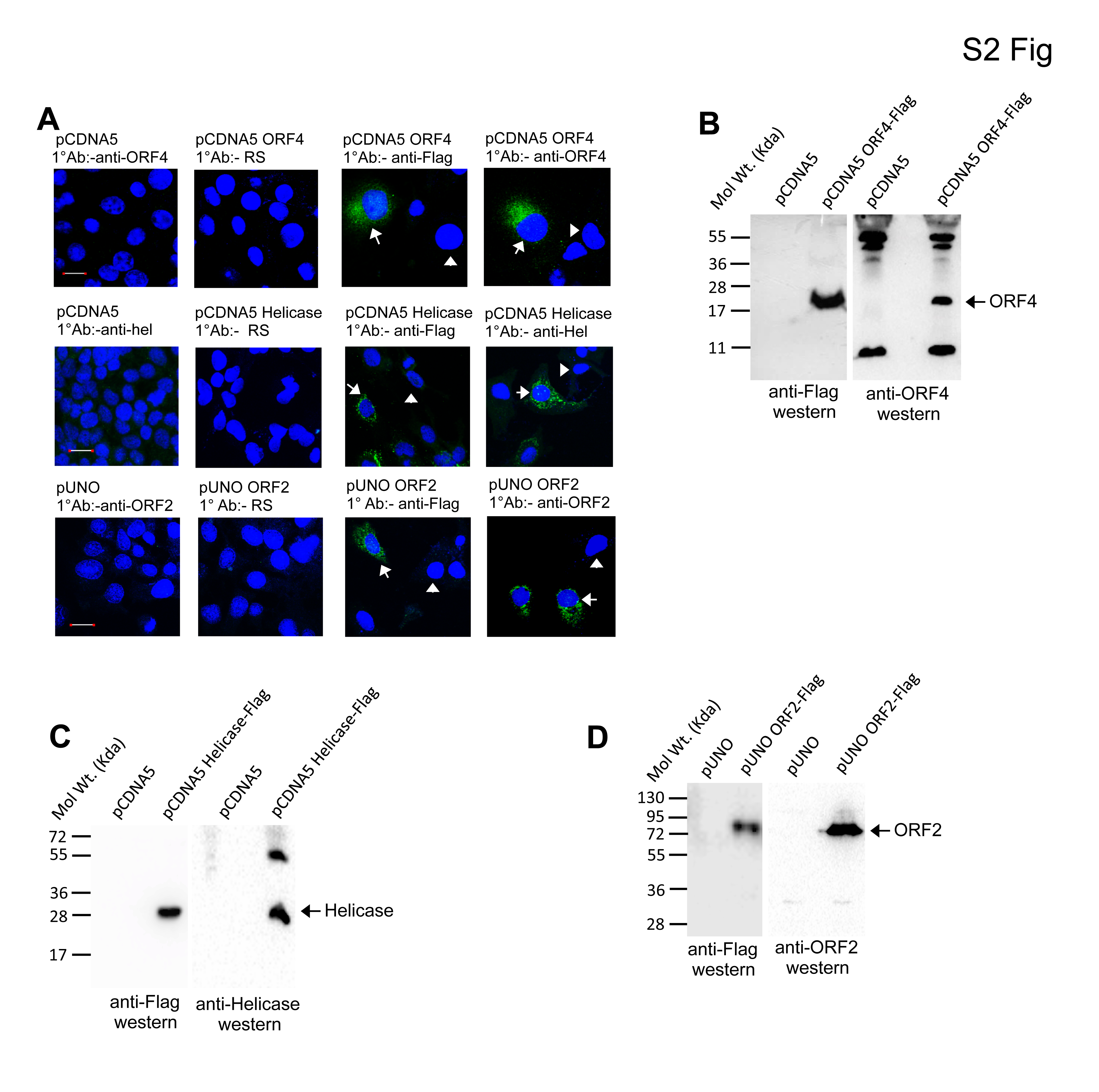

Supplement: S2 Fig — (A) Immunofluorescent visualization of ORF4 (top), Helicase (middle) and ORF2 (bottom) protein in Huh7 cells expressing respective plasmids and stained with indicated primary antibodies. “→” represents positive staining and “►”represents unstained cells. Goat anti-rabbit alexa fluor-488 secondary antibody was used for all. Nuclei stained with DAPI. RS: Rabbit pre immune serum. (B) Western of Huh7 whole cell extract transfected with pCDNA5 or pCDNA5 ORF4 plasmids using anti-Flag and anti-ORF4 antibodies, as indicated. (C) Western of Huh7 whole cell extract transfected with pCDNA5 or pCDNA5 Helicase plasmids using anti-Flag and anti-Helicase antibodies, as indicated. (D) Western of Huh7 whole cell extract transfected with pUNO or pUNO ORF2 plasmids using anti-Flag and anti-ORF2 antibodies, as indicated. (TIF) [file ppat.1005521.s002.tif]

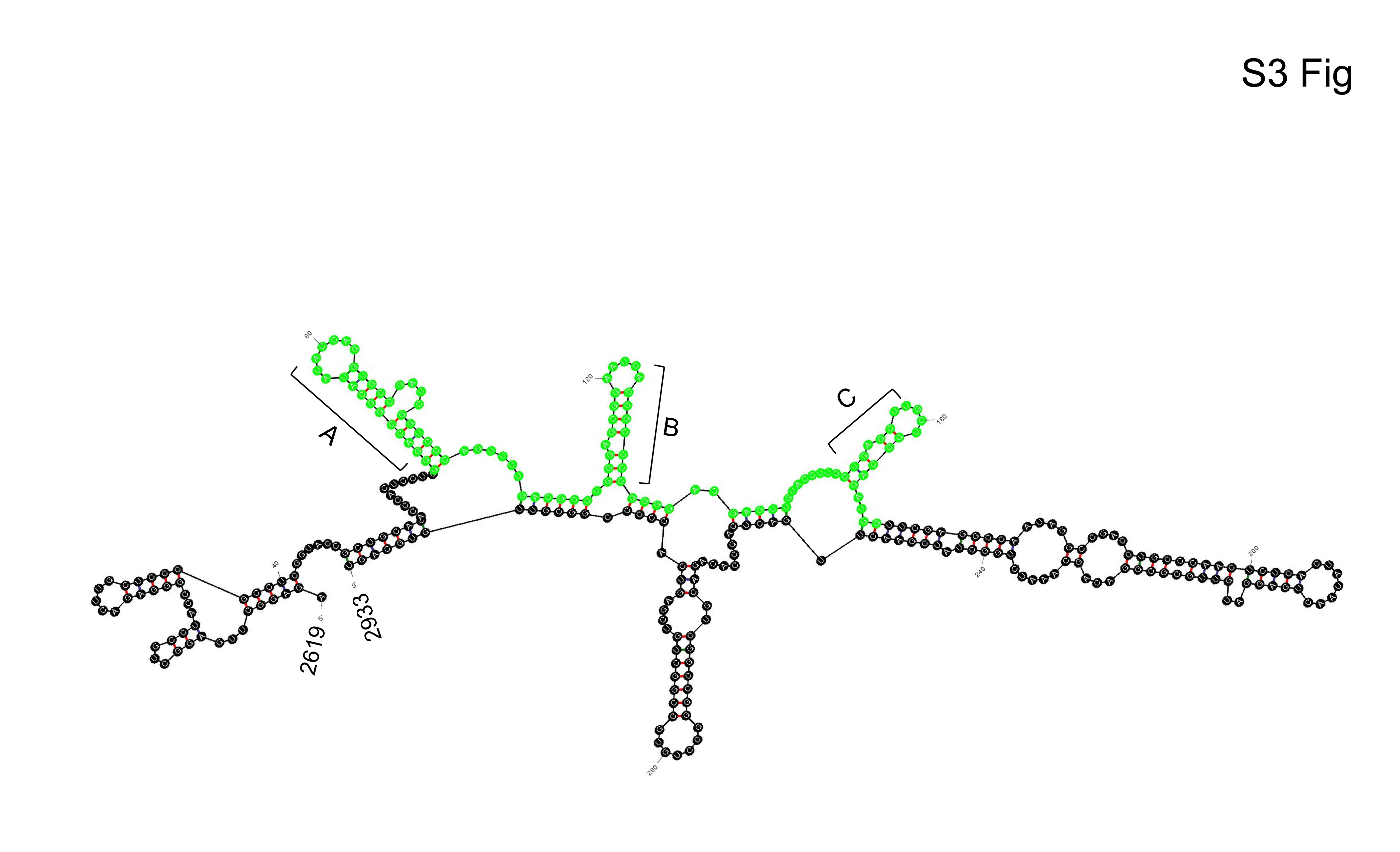

Supplement: S3 Fig — Secondary structure prediction of 2619–2933 bases (from 5’-end) of HEV genome using “mfold”. (TIF) [file ppat.1005521.s003.tif]

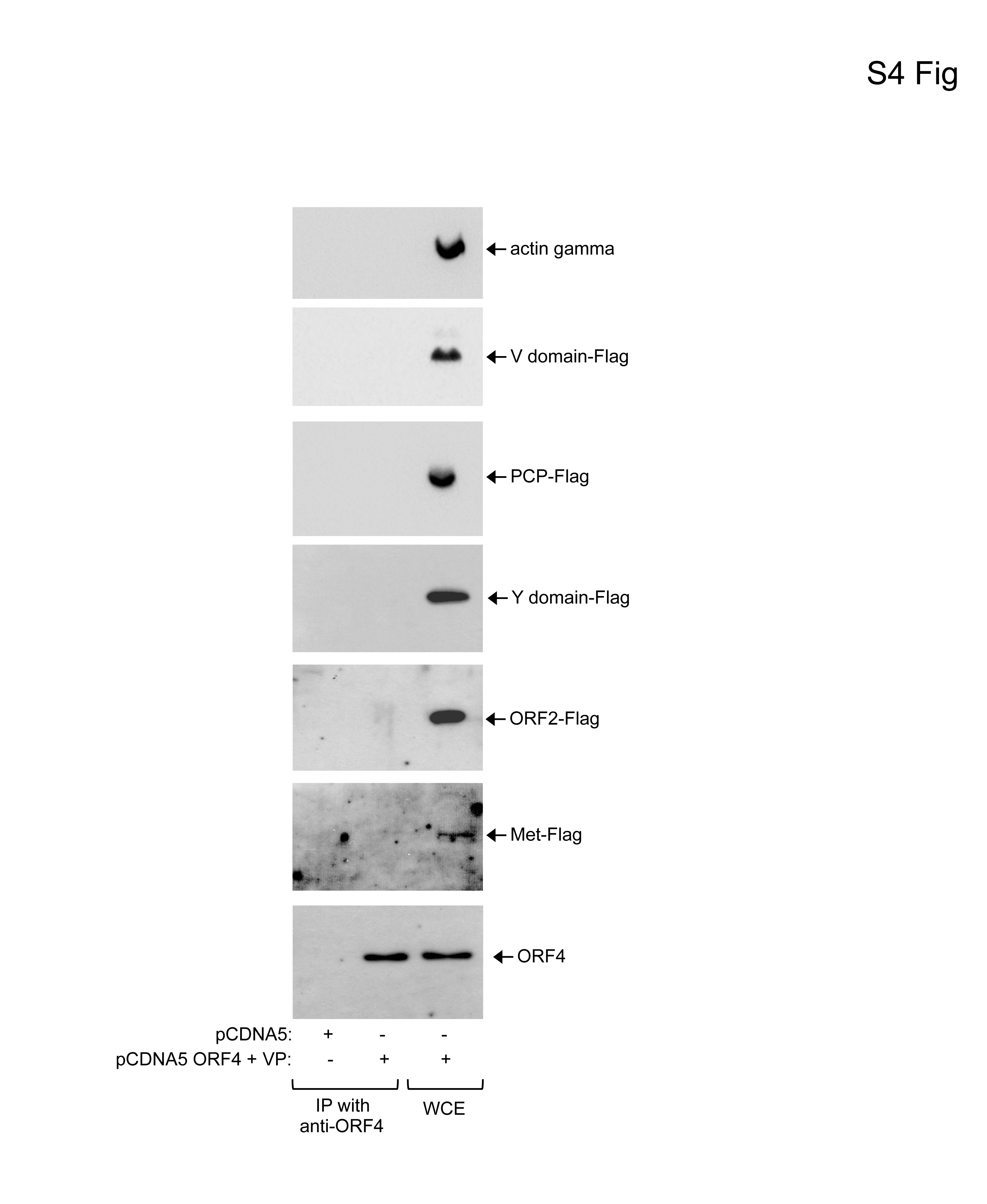

Supplement: S4 Fig — Mock (pCDNA5) or pCDNA5 ORF4 along with indicated g-1 viral protein (VP, such as Methyltransferase, ORF2, PCP, Y domain and V domain) transfected Huh7 cells were immunoprecipitated with anti-ORF4 antibody, followed by western blotting using the indicated antibodies. 25% of the pCDNA5 ORF4+VP transfected samples used for immunoprecipitation were loaded as whole cell extract (WCE). In the case of actin gamma CoIP (top panel), only pCDNA5 ORF4 was transfected. (TIF) [file ppat.1005521.s004.tif]
