# Supplementary material for: Endoplasmic Reticulum Stress Induced Synthesis of a Novel Viral Factor Mediates Efficient Replication of Genotype-1 Hepatitis E Virus
Source: PLoS Pathog. 2016 Apr 1;12(4):e1005521. doi: 10.1371/journal.ppat.1005521 (PMC4817972; doi:10.1371/journal.ppat.1005521)
Supplement: S1 Table — Summary of “ATGpr” analysis of HEV genomes. (DOCX) [file ppat.1005521.s005.docx]

**S1 Table. Analysis of HEV genomes using “ATGpr”.**

| **No. of ATG from**  **5’-end** | **Reliability^a^** | **Frame** | **Identity to Kozaq rule**  **A/GXXATGG** | **Start (base)** | **Finish (base)** | **ORF length (aa)** | **Stop codon found?** | **ORF Sequence** | **Protein product** |
| --- | --- | --- | --- | --- | --- | --- | --- | --- | --- |
| **Genotype-1 (AF444002.1)** | | | | | | | | | |
| 90 | 0.56 | 3 | AXXATGC | 5145 | 7151 | 669 | YES | MRPRPI…..DDDDKA | ORF2 |
| 3 | 0.55 | 2 | GXXATGG | 26 | 5104 | 1693 | YES | NVVHRC….ILCRVE | ORF1 |
| 87 | 0.45 | 1 | GXXATGA | 5104 | 5472 | 123 | YES | MNNMSF…QLGPRR | ORF3 |
| 50 | 0.42 | 3 | AXXATGT | 2835 | 3308 | 158 | YES | MLRGQQ…LRMYVS | Unknown (ORF4) |
| 37 | 0.35 | 2 | AXXATGG | 1826 | 5104 | 1093 | YES | MAAGPF….ILCRVE | ΔORF1 |
| **Genotype-2 (M74506.1)** | | | | | | | | | |
| 94 | 0.58 | 2 | AXXATGC | 5117 | 7093 | 659 | YES | MRPRPL…..GKTREL | ORF2 |
| 1 | 0.76 | 1 | GXXATGG | 4 | 5076 | 1691 | YES | MEAHQFI….IMHRSE | ORF1 |
| 91 | 0.60 | 3 | AXXATGA | 5084 | 5444 | 120 | YES | MNNMWF…QPGLRR | ORF3 |
| 4 | 0.52 | 2 | AXXATGA | 221 | 550 | 110 | YES | MSLSSI…RWLATA | Unknown |
| 72 | 0.36 | 1 | GXXATGG | 4060 | 5076 | 339 | YES | MVEKGQ….IMHRSE | ΔORF1 |
| **Genotype-3 (HQ389543.1)** | | | | | | | | | |
| 96 | 0.38 | 1 | AXXATGT | 5359 | 7338 | 660 | YES | MCPRVV…..GKTRES | ORF2 |
| 3 | 0.32 | 3 | GXXATGG | 27 | 5321 | 1765 | YES | MEAHQFI….IIQRVE | ORF1 |
| 93 | 0.50 | 2 | AXXATGA | 5321 | 5686 | 122 | YES | MNNMFV…QLGLRR | ORF3 |
| 47 | 0.29 | 3 | AXXATGG | 2364 | 5321 | 986 | YES | MAGYVT…. IIQRVE | ΔORF1 |
| **Genotype-4 (AJ272108.1)** | | | | | | | | | |
| 97 | 0.50 | 1 | GXXATGA | 5146 | 7161 | 672 | YES | MNNMFF…..GKTREY | ORF2 |
| 2 | 0.39 | 2 | GXXATGG | 26 | 5146 | 1707 | YES | MEAHQFI….IIYRVE | ORF1 |
| 99 | 0.50 | 2 | TXXATGG | 5174 | 5509 | 112 | YES | MEMPPC…QLGLRR | ORF3 |
| 5 | 0.46 | 3 | AXXATGA | 243 | 572 | 110 | YES | MSLSNI…QWPAMA | Unknown |
| 50 | 0.32 | 2 | AXXATGT | 2573 | 5146 | 858 | YES | MSDGFA…. IIYRVE | ΔORF1 |
| **Genotype-5 (AB573435)** | | | | | | | | | |
| 94 | 0.43 | 1 | AXXATGA | 5149 | 7170 | 674 | YES | MNNMFL…..GKTREF | ORF2 |
| 1 | 0.61 | 2 | GXXATGG | 26 | 5149 | 1708 | YES | QPRQLV….IRHRVE | ORF1 |
| 96 | 0.34 | 2 | GXXATGC | 5183 | 5518 | 112 | YES | MPPCAL…QPGLRR | ORF3 |
| 9 | 0.38 | 2 | GXXATGG | 557 | 5149 | 1531 | YES | MARHGM…. IRHRVE | ΔORF1 |
| **Genotype-6 (AB602441)** | | | | | | | | | |
| 77 | 0.45 | 1 | GXXATGA | 5152 | 7173 | 674 | YES | MNNMLS…..GKSREF | ORF2 |
| 2 | 0.47 | 2 | GXXATGG | 26 | 5152 | 1709 | YES | MEAHQFI….IVHRVE | ORF1 |
| 87 | 0.28 | 1 | GXXATGA | 5863 | 7173 | 437 | YES | MNSITS…GKSREF | ΔORF2 |
| 13 | 0.33 | 2 | GXXATGG | 557 | 5152 | 1532 | YES | MARHGM…. IVHRVE | ΔORF1 |
| **Genotype-7 (KJ496143)** | | | | | | | | | |
| 86 | 0.58 | 3 | AXXATGT | 5141 | 7151 | 670 | YES | MNNMFL…..GKTREY | ORF2 |
| 1 | 0.72 | 1 | GXXATGG | 40 | 5136 | 1689 | YES | MEAHQF….IVQRRE | ORF1 |
| 88 | 0.45 | 1 | CXXATGG | 5161 | 5499 | 113 | YES | MGTPCA…HPGLRR | ORF3 |
| 32 | 0.38 | 1 | GXXATGT | 2364 | 1840 | 5133 | YES | MSAGPY…. IVQRRE | ΔORF1 |
| 65 | 0.33 | 1 | GXXATGG | 4117 | 5133 | 339 | YES | MVEKGQ…. IVQRRE | ΔORF1 |

^a^Reliability refers to the confidence level of that prediction. “X” denotes any amino acid. N- and C- terminal sequence of each ORF is indicated in single letter code.
