# Supplementary material for: Endoplasmic Reticulum Stress Induced Synthesis of a Novel Viral Factor Mediates Efficient Replication of Genotype-1 Hepatitis E Virus
Source: PLoS Pathog. 2016 Apr 1;12(4):e1005521. doi: 10.1371/journal.ppat.1005521 (PMC4817972; doi:10.1371/journal.ppat.1005521)
Supplement: S2 Table — Summary of Yeast Two Hybrid assay using g-3 RdRp as bait and other g-3 viral proteins as prey. (DOCX) [file ppat.1005521.s006.docx]

**S2 Table. Identification of intra-viral interaction partners of g-3 RdRp by Yeast Two Hybrid assay.**

| **YEAST COTRANSFORMANTS** | **LT^-^** | **LTHA^-^** | **LTH^-^A^+^** | **LT^-^ + X-α-gal** | **LTH^-^+3AT(mM)** | | |
| --- | --- | --- | --- | --- | --- | --- | --- |
|  |  |  |  |  | **5** | **10** | **20** |
| Y2H GOLD | - | - | - | - | - | - | - |
| AD-g-3 RdRp+BD | +++ | + | + | + | + | - | - |
| AD-g-3 RdRp+BD-g-3 ORF3 | +++ | + | + | + | + | - | - |
| AD-g-3 RdRp+BD-g-3 X | +++ | + | + | + | ++ | - | - |
| AD-g-3 RdRp+BD-g-3 Helicase | +++ | + | + | + | + | - | - |
| AD-g-3 RdRp+BD-g-3 Methyl transferase | +++ | + | + | + | + | - | - |
| AD-g-3 RdRp+BD-g-3 Protease | +++ | + | + | + | + | - | - |
| AD-g-3 RdRp+BD-g-3 Y domain | +++ | + | + | + | + | - | - |
| AD-g-3 RdRp+BD-g-3 V domain | +++ | + | + | + | + | - | - |
| AD-g-3 RdRp+BD-g-3 ORF2 | +++ | + | + | + | + | - | - |
| BD-g-1 ORF3+AD-TSG101 | +++ | +++ | +++ | +++ | +++ | ++ | + |

Y2H gold strain was transformed in indicated combinations and plated on media lacking Leucine, Tryptophan (LT^-^). Eight random colonies from each transformants were replica plated to media containing various selection markers, as indicated and their growth monitored over a period of four days. Abbreviations are as in Table 1.
