# Supplementary material for: Endoplasmic Reticulum Stress Induced Synthesis of a Novel Viral Factor Mediates Efficient Replication of Genotype-1 Hepatitis E Virus
Source: PLoS Pathog. 2016 Apr 1;12(4):e1005521. doi: 10.1371/journal.ppat.1005521 (PMC4817972; doi:10.1371/journal.ppat.1005521)
Supplement: S4 Table — Summary of the Y2H cDNA library screening process. (DOCX) [file ppat.1005521.s008.docx]

**S4 Table. Screening of a human fetal brain cDNA Yeast Two Hybrid library to identify the host interaction partners of g-1 RdRp.**

A) Evaluation of self activation by the bait fusion protein.

| Two hybrid co-transformants | LT^-^ | LTH^-^ | LTHA^-^ | LTHA^-^+  A^+^ + X-αGal | LTHA^-^+ 3AT (mM) | | |
| --- | --- | --- | --- | --- | --- | --- | --- |
|  |  |  |  |  | 5 | 1O | 20 |
| pGBKT7 + pGADT7 | +++ | - | - | - | - | - | - |
| pGBKT7-RdRp +pGADT7 | +++ | +++ | +++ | ++ | + | - | - |

B) Estimation of the mating efficiency between Y2H gold (expressing the pGBKT7 RdRp, bait) and Y187 (expressing the fetal brain cDNA library in pGADT7 vector, prey) Yeast strains.

| Viral bait | Cell density of  bait culture | Cell density of prey culture | No of diploid clones screened  (in million) | Mating efficiency (in %) |
| --- | --- | --- | --- | --- |
| RdRp | 1.1*10^8^ | 3.5*10^7^ | 0.5 | 2.3 |

C) Summary of the number of RdRp interaction partners obtained at each step of the screening process.

| **Steps** | **Number of colonies and/or unique cDNA clones** |
| --- | --- |
| Number of co-transformants after replica plating on LTHA^-^A^+^ +10mM 3AT | 300 |
| Number of colonies on LT^-^+X-αgal plate after three consecutive streaking | 255 |
| Number of L^-^ plasmids isolated from Y2H gold strain | 225 |
| Number of unique clones identified by restriction pattern analysis. Clones displaying similar restriction pattern with 4 restriction enzymes were considered as one unique clone. | 174 |
| Number of L^-^ plasmids sequenced | 174 |
| Number of unique clones identified by sequencing and subsequent verification through retransformation of specific bait and prey pairs along with appropriate negative controls to monitor false positives. Includes only coding sequence, coding sequence along with 5’UTR sequence and coding sequence along with 3’UTR sequence. Clones containing sequences of hypothetical protein and uncharacterized sequences were ignored. | 33 |
| Number of unique interaction partners. Includes clones containing only coding sequence and coding sequence along with 3’UTR sequence. Clones containing coding sequence along with 5’UTR sequence were ignored. | 21 |
