# Supplementary material for: Endoplasmic Reticulum Stress Induced Synthesis of a Novel Viral Factor Mediates Efficient Replication of Genotype-1 Hepatitis E Virus
Source: PLoS Pathog. 2016 Apr 1;12(4):e1005521. doi: 10.1371/journal.ppat.1005521 (PMC4817972; doi:10.1371/journal.ppat.1005521)
Supplement: S1 File — Detailed procedure of all cloning steps and other methodologies are provided in S1 File. (DOCX) [file ppat.1005521.s009.docx]

**Supplementary methods**

**Cloning:** All primer sequences are listed in S3 Table.

pCDNA5 ORF4-Flag: ORF4 sequence was PCR amplified from pSKHEV2 using primers: pCDNA5 ORF4-flag FP and pCDNA5 ORF4-flag RP with a C-terminal FLAG epitope tag, digested with HindIII and ligated into pCDNA5 vector digested with HindIII and EcoRV.

pCDNA5 124 ORF4-Flag: 1-124 amino acids of full length ORF4 was PCR amplified from pSKHEV2 using primers: pCDNA5 ORF4-flag FP and pCDNA5 124 ORF4-flag RP with a C-terminal FLAG epitope tag, digested with HindIII and ligated into pCDNA5 vector digested with HindIII and EcoRV.

HEV 2835 mut: To mutate the first initiation codon of ORF4 (ATG-AAA), mutant fragment of 500bp was PCR amplified using primers: pGAD X FP and HEV 2835 mut RP from pSKHEV2 template, digested with MluI, AatII and ligated with gel extracted 6.9 kb and 2.9 kb fragments of pSKHEV2 vector digested with the same set of restriction enzymes.

DM HEV: A mutant of ORF4 in which both initiator ATGs are mutated to AAA was generated by digesting HEV 2835 mut with PsyI followed by gel extraction of 8.9 kb fragment which was ligated with PsyI digested mutant fragment of 1.3kb obtained by PCR using primers: HEV DM ORF4 FP and pGAD RdRp RP.

pSGI ORF4 ATG DM ORF1: DM HEV was digested with SnabI and HindIII, 4.1kb fragment gel extracted and ligated to pSGI vector digested with SnaBI and HindIII.

pSGI ORF1: ORF1 of HEV was PCR amplified using primers: HEV ORF1 FP and HEV ORF1 RP, digested with EcoRI and HindIII and ligated into pSGI vector digested with EcoRI and HindIII.

pSGI 26ATG mut ORF1: To mutate the first initiation codon of genotype-1 HEV ORF1 (ATG-CGG), mutant fragment was PCR amplified using primers: pSGI 26ATG ORF1 FP and pSGI 26ATG ORF1 RP from pSKHEV2 template, digested with HindIII and ligated into pSGI vector which was digested with HindIII and EcoRV.

26ATG mut HEV: pSGI 26ATG mut ORF1 was digested with NdeI and SnaBI, 1.5 kb fragment was gel extracted and ligated to pSKHEV2 vector digested with the same.

GAA HEV: A nucleotide at 4677 and 4680 positions were substituted with C by site directed mutagenesis to generate GDD-GAA mutations in pSK HEV2 plasmid.

GAA DM HEV: GAA HEV was digested with SfiI, XbaI, 6kb fragment gel extracted and ligated with 4kb fragment obtained by digesting DM HEV with the same enzymes.

pCDNA5 ORF4 K51N mut: Mutant fragment was PCR amplified using primers: ORF4 KN mut FP and pCDNA5 ORF4-flag RP, digested with BamHI and ligated with the 5.2kb fragment obtained by digesting pCDNA5 ORF4 with XhoI (blunted) and BamHI.

K51N HEV: K51N mutant fragment (1kb) was PCR amplified using primers: ORF4 KN mut FP, HEV ORF4 KN mut RP, digested with BamHI, SfiI. pSK HEV2 was digested with MluI, SfiI; 8.7kb fragment gel extracted. pSK HEV2 digested with MluI, BamHI; 522bp gel extracted. 1kb, 8.7kb and 522bp fragments were ligated.

IRESl mut HEV: 1.26kb IRESl mut sequence was PCR amplified using primers: IRESl mut ORF1 FP, HEV ORF4 KN mut RP; digested with PvuI, SfiI. pSK HEV2 was digested with MluI, SfiI and 8.7kb fragment gel extracted. pGAD X vector was digested with MluI, PvuI and 280 bp fragment was gel extracted. 1.2kb, 8.7kb and 280bp fragments were ligated.

pSGI IRESl mut ORF1: IRESl mut HEV was digested with SnaBI, HindIII; 4.1kb fragment gel extracted and ligated with 5.7kb fragment obtained from SnaBI, HindIII digestion of pSGI ORF1.

SL ins ORF1: Stemloop sequence fused to HEV sequence was PCR amplified from pSKHEV2 template using SL ins ORF1 FP and pGBK ORF4 RP, digested with EcoRI, SnaBI and ligated with pSGI ORF1 vector digested with same.

SL ins IRESl mut ORF1: Above mentioned EcoRI, SnaBI digested PCR product was ligated with pSGI IRESl mut ORF1 vector digested with same.

SL ins ORF1 DM ORF4: 5.8 kb was PCR amplified from DM HEV using primers: SL ins ORF1 FP and HEV ORF1 RP, digested with EcoRI, HindIII and ligated with pSGI vector digested with the same.

pRLFF/luc vector: pGL3 promoter vector (Promega) was digested with Bgl II, NcoI; blunted and self ligated. The resulting plasmid was digested with SacI, blunted, digested with BamHI and ligated into pRLTk vector digested with XbaI (blunted) and digested with BamHI.

pRLFF/luc HIRESl 315: 2619-2933 bases of genotype-1 HEV was PCR amplified from pSKHEV2 using primers HIRESl 315 FP and HIRESl 315 RP, digested with Bgl II, NcoI and ligated to pGL3 vector digested with the same. The resulting vector was digested with SacI, blunted, digested with BamHI and ligated into pRLTk vector digested with XbaI (blunted) and digested with BamHI.

pRLFF/luc HEVcRNA: pRLFF/luc HIRESl 315 was digested with AatII, blunted, digested with XhoI and 5.6 kb fragment gel extracted. pSK HEV2 was digested with SfiI, blunted, digested with XhoI and 468 bp fragment was gel extracted and ligated with 5.6kb fragment.

pRLFF/luc HIRESl87: 2701-2788 bases of genotype-1 HEV was PCR amplified using primers HIRESl 87 FP and HIRESl 87 RP, digested with XhoI, NcoI and ligated with 1.6kb and 4kb fragments obtained by digesting pRLFF/luc HIRESl 315 with the same set of restriction enzymes. Note that a KpnI site was introduced into the vector through the reverse primer.

pRLFF/luc HIRESl87 mut A, B, C, A*, AC, BC: Primers incorporating the altered bases were used to PCR amplify 2701-2787 bases of genotype-1 HEV from pSKHEV2. PCR product was digested with XhoI and KpnI and ligated with pRLFF/luc HIRESl87 digested with same. Following primer pairs were used: mut A: HIRESl 87 mut A FP, HIRESl 87 RP, mut B: HIRESl 87 mut B FP, HIRESl 87 RP, mut C: HIRESl 87 FP, HIRESl 87 mut C RP, mut A*: HIRESl87 mut A* FP, HIRESl 87 RP, mut AC: HIRESl 87 mut A FP, HIRESl 87 mut C RP, mut BC: HIRESl 87 mut B FP, HIRESl 87 mut C RP.

pGEX4T-1- ORF4: ORF4 sequence was PCR amplified from pGBKORF4 template using primers: GST-ORF4 FP, pCDNA5 ORF4-flag RP; digested with EcoRI and ligated into pGEX4T-1 vector digested with XhoI (blunted) and EcoRI.

pUNO X-HA: X sequence was PCR amplified from pSKHEV2 template using primers: pUNO X-HA FP, pUNO X-HA RP; digested with BamHI, NheI and ligated into pUNO vector digested with same.

pVITRO2 RdRp-myc: RdRp sequence was PCR amplified from pGBKT7 RdRp using primers: pVITRO RdRp-myc FP, pGAD RdRp RP; digested with XhoI and ligated into pVITRO2 vector digested with EcoRI and Sal I.

pVITRO2 ORF3-HA: ORF3 sequence was PCR amplified using primers pVITRO ORF3-HA FP, pVITRO ORF3-HA RP; digested with BamHI, Sal I and ligated into pVITRO2 vector digested with Bgl II and XhoI.

pUNO ORF2-Flag: ORF2 with C-terminal Flag tag was PCR amplified with primers: pUNO ORF2-flag FP, pUNO ORF2-flag RP; digested with Bgl II and ligated into pUNO vector digested with NheI (blunted) and BamHI.

pCDNA5 Helicase-Flag: Helicase sequence was PCR amplified using primers: pCDNA5 HEL-flag FP, pCDNA5 HEL-flag RP; digested with HindIII and ligated into pCDNA5 vector digested with HindIII and EcoRV.

pUNO RdRp-flag: RdRp sequence with C-terminal flag tag was PCR amplified, digested with AgeI, NheI and ligated into pUNO vector digested with same.

pUNO Helicase-myc: Helicase sequence with C-terminal myc tag was PCR amplified, digested with AgeI, NheI and ligated into pUNO vector digested with same.

pUNO g-3 RdRp-flag: Genotype 3 HEV RdRp sequence with C-terminal flag tag was PCR amplified from pSK p6 luc template, digested with AgeI, NheI and ligated into pUNO vector digested with same.

pUNO g-3 X-HA: Genotype 3 HEV X domain sequence with C-terminal HA tag was PCR amplified from pSK p6 luc template, digested with AgeI, NheI and ligated into pUNO vector digested with same.

pUNO g-3 Helicase-myc :Genotype 3 HEV helicase sequence with C-terminal myc tag was PCR amplified from pSK p6 luc template, digested with AgeI, NheI and ligated into pUNO vector digested with same.

pGBK ORF4: ORF4 sequence was PCR amplified from pSK HEV2 using primers: pGBKORF4 FP, pGBKORF4 RP; digested with NdeI, EcoRI and ligated into pGBKT7 vector digested with same.

pGAD ORF4: pGBK ORF4 was digested with NdeI and EcoRI and ligated into pGAD vector digested with same.

pGAD 1-41 ORF4: pGAD ORF4 was digested with BamHI and the 8.1kb fragment was gel extracted and religated.

pGAD 1-61 ORF4: pGAD ORF4 was digested with SmaI and the 8.1kb fragment was gel extracted and religated.

pGAD 1-124 ORF4: pGAD ORF4 was digested with ClaI and the 8.4kb fragment was gel extracted and religated.

pGAD 54-105, pGAD 54-122 and pGAD 76-122 amino acid deletion mutants of full length ORF4 was PCR amplified (primers in Table S3), digested with NdeI, EcoRI and ligated into pGADT7 vector digested with same.

pGAD RdRp, pGAD methyl transferase, pGAD protease, pGAD X, pGAD Y and pGAD V was PCR amplified using corresponding primers, digested with EcoRI, XhoI and ligated into pGADT7 vector digested with same.

pGAD Helicase: Helicase sequence was PCR amplified from pSKHEV2 with primers: pGAD hel FP, pGAD hel RP; digested with NdeI, EcoRI and ligated into pGADT7 vector digested with same.

pGBK RdRp, pGBK methyl transferase, pGBK protease, pGBK X, pGBK Y and pGBK V: pGAD RdRp, pGAD methyl transferase, pGAD protease, pGAD X, pGAD Y and pGAD V was digested with EcoRI, XhoI and ligated into pGBKT7 vector digested with EcoRI and SalI.

pSUPER heEF1α1: 2 sets of oligos targeting two different regions of heEF1α1 messenger was commercially synthesized (heEF1α1 shRNA U and L). Upper and lower strand specific oligos were phosphorylated at 5’-end, annealed and ligated into pSUPER puro vector digested with Bgl II and HindIII.

pSUPER hTubβ: 2 sets of oligos targeting two different regions of hTubβ messenger was commercially synthesized (hTubβ shRNA U and L). Upper and lower strand specific oligos were phosphorylated at 5’-end, annealed and ligated into pSUPER puro vector digested with Bgl II and HindIII.

pSUPER EGFP: Oligo targeting EGFP messenger was commercially synthesized (EGFP shRNA U and L). Upper and lower strand specific oligos were phosphorylated at 5’-end, annealed and ligated into pSUPER puro vector digested with Bgl II and HindIII.

**Cell culture, transfection and shRNA expression**: Huh7 cells were maintained in Dulbecco’s modified Eagle medium (DMEM) containing 10% Fetal Calf Serum (FCS), 50 I.U./mL Penicillin and Streptomycin, in 5% CO_2_. Cells were transfected using Lipofectamine 2000 or 3000, following manufacturer’s protocol (Life Technologies, USA). For generating ORF4-huh7 and pCDNA5-huh7 cell line, pCDNA5 ORF4 and pCDNA5 vectors were linearized with XhoI, transfected into huh7 cells, followed by hygromycin treatment (200μg/ml). Clonal population of stable transfectants were enriched by serial dilution and four clones selected for further expansion. 6μg HEV genomic RNA was transfected into Huh7 cells in 6 well plates at 70% confluency using lipofectamine 3000. 24 hours post transfection, culture medium was changed. Cells were harvested on 6^th^ day. 2μg g-3 HEV genomic RNA (pSK HEV p6 luc) was electroporated into 1.5ₓ10^6^ huh7 cells (200V, 950μF, ∞). Five days later, cells were harvested for measuring cell viability using WST-1 reagent and culture medium was used for luciferase assay.

3μg DNA of each pSUPER construct expressing shRNAs against EGFP, heEF1α1 or hTubβ were transfected into Huh7 cells on 60mm TC dish at 70% confluency using lipofectamine 2000. 24 hours post transfection, culture medium was changed and 1μg/ml Puromycin was added every alternate day. Cells were harvested on 7^th^ day, whole cell extract prepared and western blotted with specific antibodies to monitor the level of respective proteins. Of the two clones of heEF1α1 and hTubβ, clones that were more effective in reducing the level of respective proteins, were selected for future experiments. For transfecting viral RNA or expression plasmids into shRNA expressing cells, on day 7 post-transfection and Puromycin selection, cells from multiple dishes were pooled and reseeded at 70% confluency overnight, followed by transfection of respective DNA or RNA, as mentioned above. Prior to proceeding with various experiments, an aliquot of the cells were western blotted with anti-eEF1α1 or anti-Tubβ antibodies to monitor the level of respective proteins.

**Generation and validation of antibodies:** Rabbit polyclonal antibodies against helicase, ORF2 and ORF4 proteins of HEV were generated at Genscript (USA) using KLH (Keyhole Limpet hemocyanin) conjugated peptide antigens. Peptide sequences are: Helicase: PTRELRNAWRRRGFC, ORF2: QQDKGIAIPHDIDLC, ORF4: CLESPRGAGLSLMRP. All peptides were HPLC purified to >98% purity. Pre immune serum was collected from the animals prior to immunization. Antibodies were affinity purified and stored in PBS (pH-7.4) with 0.2% sodium azide. ELISA assays using peptides as antigen and pre immune serum as negative controls confirmed their titres to be > 1:512,000. In competition assays, corresponding peptides could block the binding of cognate antibodies. Functionality of these antibodies in western and immunofluorescence assays were validated by testing their ability to detect corresponding proteins expressed in Huh 7 cells. For immunofluorescence, C-terminal flag-tagged Helicase, ORF2 and ORF4 expression constructs were transfected into Huh7 cells on coverslips and incubated with anti-flag or anti-helicase, anti-ORF2, anti-ORF4 antibodies (1:1000 dilution for anti-flag, anti-helicase and anti-ORF2; 1:200 dilution for anti-ORF4) or pre immune serum (1:200 dilution) in parallel. Empty vector expressing cells were used as negative controls. Goat anti-rabbit alexa fluor-488 was used as secondary antibody. All three antibodies specifically recognized the corresponding proteins and localization of proteins were similar to that obtained with anti-flag antibody (S2A Fig). For western blotting, Huh 7 cells were transfected with empty vector or flag-tagged helicase, ORF2 and ORF4 expressing constructs, followed by western blotting of whole cell extract using anti-flag (1:1500), anti-ORF2 (1:1000), anti-helicase (1:1000) and anti- ORF4 (1:1000) antibodies (S2B, S2C, S2D Fig). All antibodies produced expected size bands, confirming their specificity.

**Immunofluorescence assay:** Huh-7 cells were seeded into 6-well culture dish containing glass coverslips at 50% confluency and maintained in antibiotic free DMEM supplemented with 10% FBS. 1µg plasmid DNA was transfected into each well using Lipofectamine 2000 in optiMEM. 24 hours post transfection, fresh culture medium was added and incubated for another 24 hours. 48 hours post transfection, cells were washed once in PBS at ambient temperature, followed by fixation in 2mL 100% methanol for 15 minutes at -20^0^C and washed thrice in 2mL PBS. Coverslips were incubated with blocking solution (5% Normal Goat Serum, 0.5% BSA in 1X PBS, 0.3% Triton-X-100) for 60 minutes at room temperature followed by single wash in PBS and incubation with primary antibodies (in 1X PBS,0.3% Triton-X-100, 5% BSA) overnight at 4^0^C. Primary antibody dilutions were:- αFlag- 1:1000, αORF2- 1:1000, αhelicase- 1:1000, αORF4- 1:200, Pre immune serum- 1:200. Next day, coverslips were washed thrice with PBS and incubated for 1 hour with 1:500 dilution of goat anti-rabbit alexa fluor-488 (in 1X PBS, 0.3% Triton-X-100, 5% BSA). Next, coverslips were washed thrice in PBS and mounted on glass slides using antifade gold (Molecular probes, USA), which also contains DAPI (4’, 6’-diamino 2-phenyl indole). Images were acquired using a 60X objective in a confocal microscope (Olympus FV1000) and analyzed by Fluoview software.

For immunofluorescence visualization of HEV proteins expressed by the viral genome, 6µg in vitro synthesized genomic RNA was transfected into Huh7 or S10-3 cells at 70% confluency in 6-well tissue culture dishes using Lipofectamine 3000/2000. Medium was replaced 24 hour post transfection. 4^th^ day post transfection, _˜_95% confluent cells were seeded into 6-well tissue culture dish containing coverslips at 50% confluency. 24 hours post seeding, required compounds were added and incubated for indicated period, followed by washing in PBS and downstream processing as described above for plasmid transfected cells.

**Immunoprecipitation and western blotting:** ~95% confluent cells were washed once in PBS at room temperature followed by addition of 800µl CoIP buffer [20mM Tris (pH 7.4), 150mM NaCl, 1mM EDTA (pH 8.0), 1mM EGTA (pH 8.0),, 1% Triton X 100, 2.5mM Sodium Pyrophosphate, 1mM β glycerol phosphate, 1mM sodium orthovanadate, protease inhibitor cocktail] and scrapping into 1.5ml tube on ice. A homogenous suspension was generated by repeated pipetting and vortexing, followed by overnight incubation on ice. Next day, lysate was centrifuged at 13000g, 4°C, 10’ and supernatant collected into fresh tube. Protein amount was equalized by Bradford assay. If same lysate had to be used for western blotting, an aliquot was taken, mixed with 3X Laemelli buffer [187.5mM Tris (pH 6.8), 6% SDS, 30% glycerol, 150mM DTT and 0.03% Bromophenol blue] and incubated at 95°C for 5 minutes. For immunoprecipitation or Coimmunoprecipitation assay, equal amount of lysate was incubated with desired antibodies (anti-HA, Flag and Myc antibodies: 1µg each; anti-helicase: 4µg) at 4°C overnight on a rocker, followed by incubation with 30µl Protein A magnetic beads (Cell Signalling, USA) for 1 hour, under similar condition. Protein A bound proteins were washed three times in CoIP buffer and eluted by incubating in Laemelli buffer at 95°C for 5 minutes.

For western blotting, proteins were resolved by SDS-PAGE, transferred to PVDF (Polyvinylidene fluoride) membrane (Pall Corp, USA) and blocked using 5% skimmed milk or BSA, incubated with primary and secondary antibodies. Bands were revealed by enhanced chemiluminiscence using a commercial kit (Biorad, USA). Images were acquired and analyzed using a gel documentation system (Chemidoc MP, Biorad, USA).

**RNA strand displacement Assay:** Two RNA oligos: HRNA1 5’-UUUUUUUUUUUUCGCUGAUGUCGCCUGG and HRNA2 5’-[6FAM] CCAGGCGACAUCAGCG were synthesized commercially (Sigma, USA). 5 pmol denatured HRNA1 was resolved by 20% PAGE followed by Ethidium Bromide to monitor its integrity. 5 pmol denatured HRNA2 was resolved in 20% PAGE, followed by visualization of fluorescence at 488nm using a gel documentation system (Chemidoc MP, Biorad, USA). Oligos were diluted in 10mM Tris-Cl (pH 7.4), 100mM NaCl, 1mM EDTA; incubated at 95°C for 2 minutes, followed by gradual cooling to room temperature overnight. Annealed RNA duplex were resolved in 20% non-denaturing PAGE and band corresponding to double stranded RNA was excised, minced and soaked in 500μl soaking buffer (0.5M Ammonium acetate, 10mM EDTA and 1% SDS) overnight on rocker at room temperature. Next day, samples were centrifuged at 13000g for 5 minutes at room temperature. Supernatant was transferred to a fresh microcentrifuge tube, 5μg glycogen and 1mL ice cold absolute ethanol was added and incubated at -80°C for 45 minutes, followed by centrifugation at 14000g for 30 minutes at 4°C. Pellet was dissolved in 30μl nuclease-free water, incubated at 55°C for 10 minutes, quantitated on gel as well as by spectrophotometry, aliquoted and stored in -80°C.

10 pmol dsRNA, 2µg Flag-affinity purified HEV helicase protein or HEV ORF2 protein, increasing amount of GST-ORF4 protein (100-300ng) were incubated in strand displacement buffer [50mM HEPES(pH-7), 2mM MgCl_2_, 10mM KCl, 0.05mg BSA/mL, 2mM DTT, 1mM ATP] at 37°C for 2 hours. The reaction was terminated using 0.2%SDS and 20mM EDTA, followed by proteinase K (20µg) treatment for 10 minutes at 37°C. 5µg glycogen and 1mL of absolute ethanol was added to the samples, incubated at -80°C for 45 minutes, centrifuged at 14000g, 30 minutes, 4°C. Pellet was washed with 1ml 75% ethanol and resuspended in 10µl water. Samples were mixed with RNA loading dye (Thermo Scientific, USA), resolved in 20% PAGE and fluorescent bands imaged, as described above.

An equal amount of dsRNA containing sample was heat denatured (95°C, 10 minutes) immediately before loading in the gel to differentiate the migration between single strand (ss) RNA and dsRNA (denoted by **_#_** in Fig 7C). ORF4 and ORF2 were used as negative controls to demonstrate specificity of the helicase used in the assays.

For performing helicase assay using Huh7 cell purified ORF4-flag, ORF4-flag was purified by Flag-affinity method, quantitated by Bradford assay and silver staining. 2µg each of helicase, ORF4 or ORF2 was used for the assay in the indicated combination.

**RNA dependent RNA polymerase assay (RdRp assay):** HEV RdRp was Flag affinity purified for use in RdRp assay. Similarly purified ORF2, ORF4 or 124 ORF4 were also used. HEV genomic regions 1-130 bases and 6995-7204 bases (from 5’-end) were fused to each other by removing 131- 6994 bases from pSKHEV 2 plasmid. This plasmid was linearized using Bgl II and in vitro transcribed using mMessage mMachine T7 kit (Ambion, USA) to generate a 340 nucleotide long capped RNA, which was used as template in RdRp assays to monitor antisense strand synthesis. Size and integrity of the RNA was verified by formaldehyde agarose gel electrophoresis (Fig 6H). RNA was quantified by spectrophotometry and stored in single use aliquots at -80°C.

0.5µg RNA was mixed with 1, 5, 10µg RdRp or 5µg ORF2 and 100, 200 or 300ng GST-ORF4 or 5µg ORF4-flag in assay buffer containing 200mM Tris (pH 7.5), 100mM MgCl_2,_ 200mM DTT, 200mM KCl, 100mM EDTA (pH 8.0), 500µM ATP, 500µM CTP, 500µM GTP, 250µM UTP, 250µM DIG-11-UTP, 2µg Actinomycin-D and 40 units RNAsin in a total volume of 40µL and incubated at 22°C for 2 hours. In case of RNase A digested sample, 10µg RNase A was added after 2 hours for 10 minutes at 37°C. Next, all samples were treated with 50µg Proteinase K in PK buffer [300mM NaCl, 100mM Tris-Cl (pH 7.5), and 1% SDS] for 30 minutes at 37°C. Next, 5µg glycogen and 1ml absolute ethanol was added to the samples, incubated at -80°C for 1 hour, centrifuged at 14000g, 15 minutes, 4°C and supernatant was removed. Pellet was washed in 1ml 75% ethanol and resuspended in 10µl water. These samples were resolved by 2% formaldehyde agarose gel electrophoresis, as instructed [38]. RNA molecular weight marker (Millennium marker, Ambion, USA) was run in parallel to monitor the size of the RdRp assay products. Lane corresponding to marker was excised and stained with Ethidium bromide to visualize the markers. Rest of the gel was proceeded for transfer to Nylon membrane, as described [38]. Nylon membrane was UV-crosslinked and rinsed in DEPC water followed by incubation with wash buffer (100mM Maleic acid, 150mM NaCl, 0.3% Tween-20, pH 7.5) for 5 minutes at room temperature. Next, membrane was blocked in 1X blocking solution (from Northern starter kit, Roche, USA) for 30 minutes at room temperature, incubated with anti-DIG primary antibody (Roche, 1:10,000 dilution in blocking solution) for 30 minutes at room temperature, washed twice in wash buffer, equilibrated with detection buffer (100mM Tris-Cl, 100mM NaCl, pH 9.5) for 5 minutes at room temperature and developed using CDP-star. Chemiluminiscent images were acquired using a gel documentation system (Chemidoc MP, Biorad, USA) or by autoradiography.
